# Enhancing water access monitoring through mapping multi-source usage and disaggregated geographic inequalities with machine learning and surveys

**DOI:** 10.1038/s41598-023-39917-6

**Published:** 2023-08-18

**Authors:** Jan Geleijnse, Martine Rutten, Didier de Villiers, James Tayebwa Bamwenda, Edo Abraham

**Affiliations:** 1 Department of Water Management, Delft University of Technology, Mekelweg, 2628 CD Delft, The Netherlands; 2 UNICEF, Nairobi, Kenya; 3 Makerere University, University Rd., P. O. Box 7062, Kampala, Uganda

**Keywords:** Environmental sciences, Engineering, Environmental social sciences, Climate-change adaptation, Psychology and behaviour, Socioeconomic scenarios, Mathematics and computing, Information technology

## Abstract

Monitoring safe water access in developing countries relies primarily on household health survey and census data. These surveys are often incomplete: they tend to focus on the primary water source only, are spatially coarse, and usually happen every 5-10 years, during which significant changes can happen in urbanisation and infrastructure provision, especially in sub Saharan Africa. In this work, we present a data-driven approach that utilises and compliments survey based data of water access, to provide context-specific and disaggregated monitoring. The level of access to improved water and sanitation has been shown to vary with geographical inequalities related to the availability of water resources and terrain, population density and socio-economic determinants such as income and education. We use such data and successfully predict the level of water access in areas for which data is lacking, providing spatially explicit and community level monitoring possibilities for mapping geographical inequalities in access. This is showcased by applying three machine learning models that use such geographical data to predict the number of presences of water access points of eight different access types across Uganda, with a 1km by 1km grid resolution. Two Multi-Layer-Perceptron (MLP) models and a Maximum Entropy (MaxEnt) model are developed and compared, where the former are shown to consistently outperform the latter. The best performing Neural Network model achieved a True Positive Rate of 0.89 and a False Positive Rate of 0.24, compared to 0.85 and 0.46 respectively for the MaxEnt model. The models improve on previous work on water point modeling through the use of neural networks, in addition to introducing the True Positive - and False Positive Rate as better evaluation metrics to also assess the MaxEnt model. We also present a scaling method to move from predicting only the relative probability of water point presences, to predicting the absolute number of presences. To challenge both the model results and the more standard health surveys, a new household level survey is carried out in Bushenyi, a mid-sized town in the South-West of Uganda, asking specifically about the multitude of water sources. On average Bushenyi households reported to use 1.9 water sources. The survey further showed that the actual presence of a source, does not always imply that it is used. Therefore it is no option to rely solely on models for water access monitoring. For this, household surveys remain necessary but should be extended with questions on the multiple sources that are used by households.

## Introduction

Achieving universal access to safe and affordable drinking water for all is fundamental for human well-being and sustainable development, as set out by the United Nations Sustainable Development Goals—specifically SDG 6.1^1^. Although international surveying of drinking water and sanitation access goes back decades^2^, the monitoring of SDG targets is becoming an essential input in the design and assessment of infrastructure development plans, as well as policy on water access and other infrastructure services.

The official monitoring of SDG6 lies in the hands of the Joint Monitoring Program (JMP), which is a collaboration of UNICEF and the World Health Organisation (WHO). This monitoring relies primarily on household health surveys and census data^2^. However, there are some limitations to the approach: firstly, survey questions on water access typically focus on the primary drinking water source only. In reality, in developing countries with low scores for SDG Target 6.1, people often use multiple water sources for different purposes, including for drinking^3,4^. Such a focus only on the primary drinking water source can therefore overestimate the population share with permanent safe access, since some of the other sources used may not be safe^4^. Secondly, health survey data is spatially coarse. For example, in Uganda’s Demographic Health Survey (DHS), households that are 10 kilometres apart can be clustered in the same group for privacy reasons^5^, wherein there might be large differences in access levels. Thirdly, health surveys are repeated every 5 to 10 years, during which time rapid urbanisation processes can change the water access situation significantly. The JMP makes progress updates based on available recent statistical data at country, regional and global levels^6^, which are very useful for tracking access targets and making inter-country comparisons. Nonetheless, these are not sufficiently detailed for decision making on infrastructure sub-nationally and do not account for multiple water source use. Especially in sub-Saharan Africa (SSA), where access levels are low and geographically quite heterogeneous, there is an urgent need for a spatially explicit and community level monitoring to accelerate progress towards meeting SDG targets by 2030.

**Figure 1:**
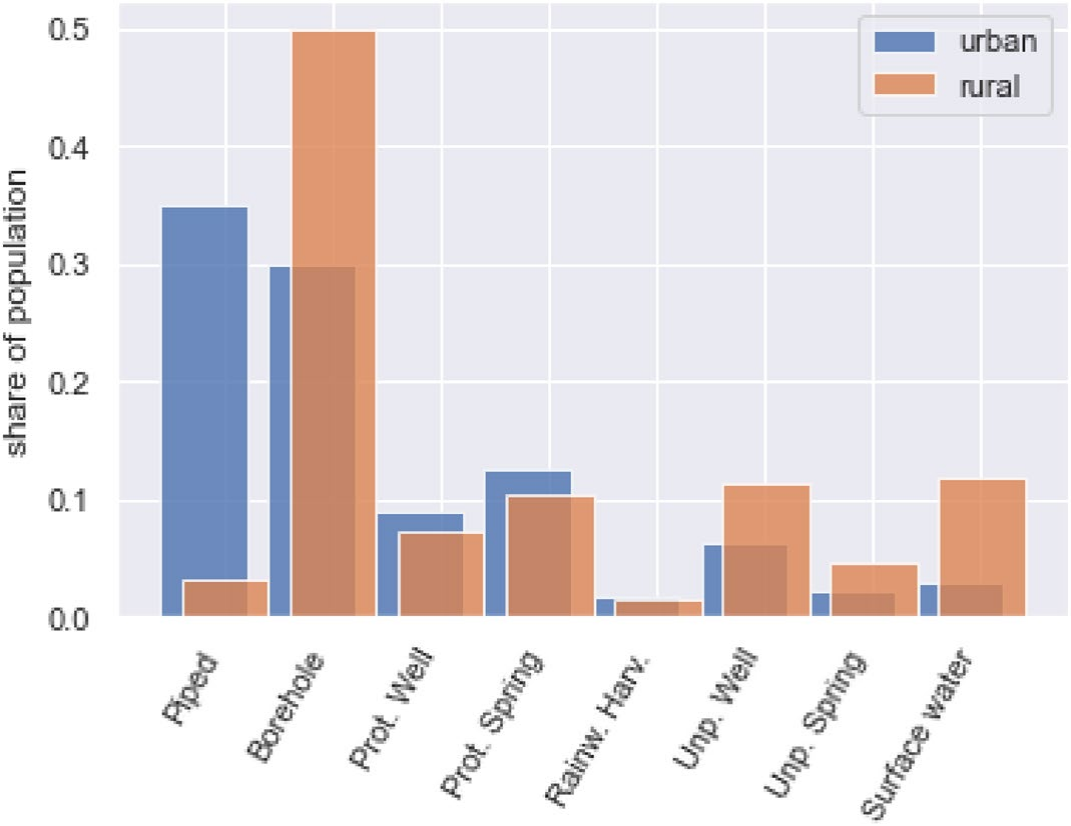
Share of population using the various access types in Uganda. Figure made from the Uganda Demographic and Health Survey 2016 data^5^.

**Figure 2:**
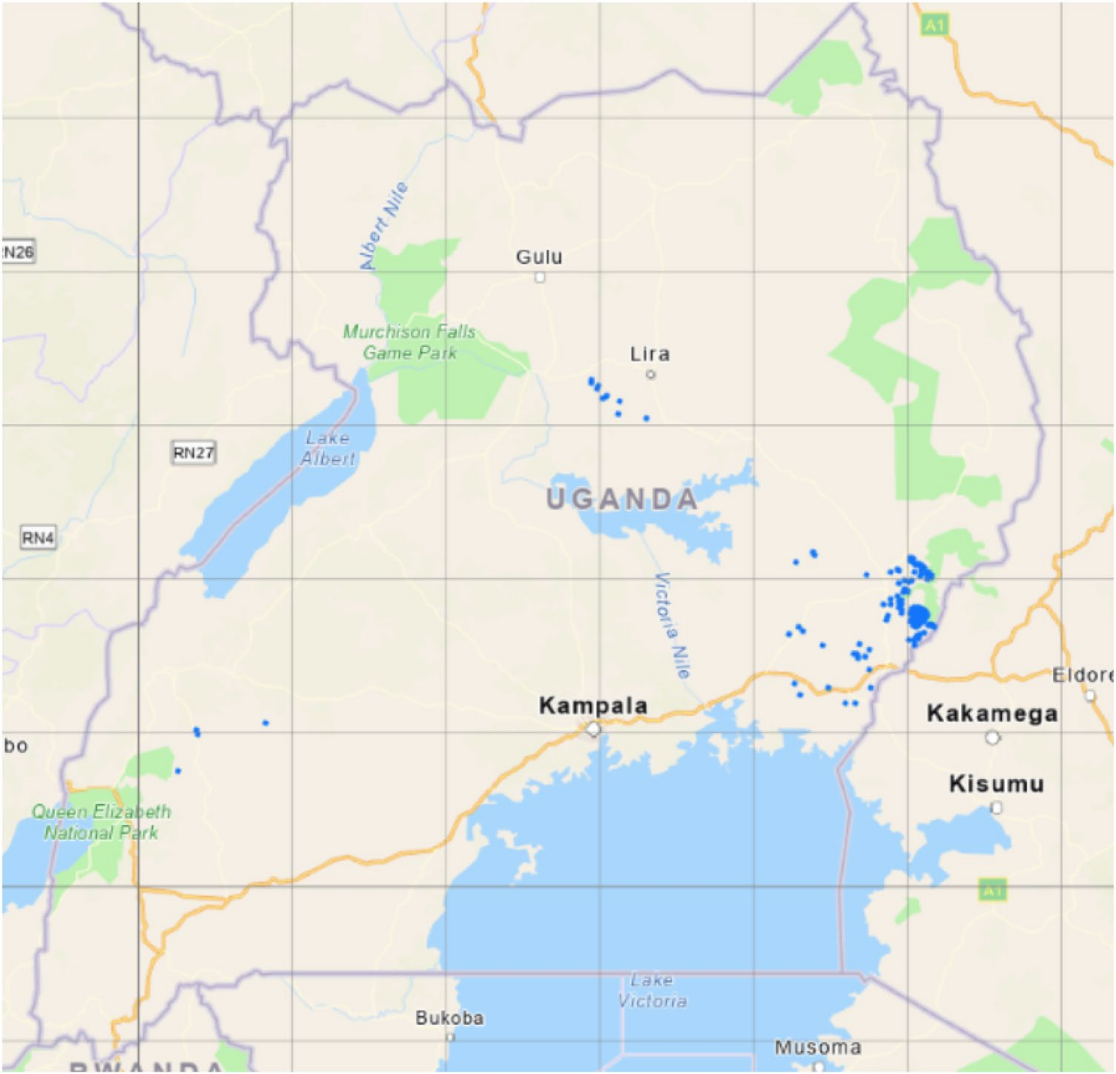
WPDx coverage for unprotected springs (blue dots) in Uganda. Figure made using ArcGis Pro (TU Delft subscription 6423753137).

The level of access to improved water and sanitation has been shown to vary with geographical inequalities related to socio-economic determinants such as income and education, and also based on the availability of water resources^7–10^. These differences are also typified by the stark differences in urban and rural access levels in SSA. For instance, according to JMP in Uganda, 48% versus 76% of the population accesses at least basic drinking water in rural and urban areas respectively. In more detail in Fig. 1, the differences between the water sources used is large for urban and rural data. However, there is not much information besides the urban rural distinction. The increased availability of sufficiently detailed geographical data-sets on such socio-economic and geohydrological parameters brings the potential to use big data to monitor the progress on SDG Target 6.1 in more (spatial) detail. In addition, geospatial water infrastructure databases like the Water Point Data Exchange (WPDx) are now available, which enables collecting of water access points and their status with a digital platform on which NGOs, governments and water companies can share such data. Water point data consist of the geographical coordinates of a water access point and the water access type (borehole, spring, etc.) and are available through http://www.waterpointdata.org. Unlike health surveys, and water access and sanitation levels based on these surveys^9,10^, these data sets could better capture the multitude of different types of sources used in an area. A limitation of using only WPDx for monitoring access is that the presence of a source does not always mean it is actually used^3,4^—as it goes for many infrastructure services, availability is not equivalent to access for all. Besides, as can be seen in Fig. 2 for Uganda, coverage of the WPDx database is also not extensive at the moment or equally distributed nationally.

To overcome the limited coverage of access point data, costly investments are needed in systematic collection of more WPDx data. Another option, suggested before^11^ and in this manuscript, is to apply modeling methods that can predict the presence of water access points in areas for which we do not have water point data. An additional advantage of such models is that they could also give insight into determinants of water access and access types, and with that why access is higher in some areas or communities versus others.

Building such predictive models is a difficult task given that WPDx data is ‘presence only’ data, with no additional information on non-surveyed areas on whether they do or do not have water access points (see Section "Presence only data"). Previous work has applied a Bayesian biological species modeling technique called MaxEnt, which is well suited for modelling with presence only data, to model the relative probability distribution of finding surface water access points and unimproved wells across Kenya from parts of the WPDx data for Kenya^11^. Their results were promising judging by the predictive power of the models that score 0.9 at the Area Under the Receiver Operator Curve (AUC, a common evaluation parameter for such classifying models). The model uses a number of physical, geographical and socio-economical features (predictive data) of which the model learned the combination of characteristics that may have predictive power on the presence of water access points^12^.

In a presence only model comparison study for species modelling, it was shown that machine learning models such as MaxEnt outperform the more traditional regressive models for such data^13^. A different study even showed that MaxEnt performs almost just as well on presence only data as regressions on presence-absence data, even though presence-absence data sets are expected to allow for better comparison^14^. However, in earlier work^15^, MaxEnt was compared to the performance of neural network models for the distribution of presence of five plant types in France and it was shown that the neural network models can outperform MaxEnt, mainly because they are able to capture more complex nonlinear transformations and combinations of input features better than MaxEnt. Neural networks have been shown to be most capable of modeling very non-linear processes with large set of socio-economic and environmental predictive features such as in forecasting energy prices^16^ and water prices^17^.

This paper uses both survey- and data-driven monitoring techniques. By adapting species modeling techniques to use WPDx and other data of access determinants, we show how the increasingly widely available socio-economic and geohydrological data can be used to improve on nationwide and local water access level monitoring; the models presented in this manuscript estimate the number of water access points in Uganda with a resolution of one square kilometer. The models improve on earlier water point modeling work^11^ by applying neural networks next to a MaxEnt model, by presenting a scaling method to move from relative probability to the absolute number of presences and by presenting a weighted background selection of pseudo-absence locations. As also demonstrated through administrative region level disaggregation in Ref.^7^, this approach could potentially support a spatially explicit higher resolution mapping of geographic inequalities in water access that goes beyond the usual rural-urban divide and national level reporting.

In addition to the predictive models, a household survey campaign was carried out in Bushenyi, a mid-sized town with both rural and urban characteristics in the South-West of Uganda. The focus of the survey is on the multitude of sources that households make use of, providing detailed data on the actual usage of different water sources, which is then used to challenge the data driven models. The findings indicate that the presence of a source does not always mean it is actually used, e.g. we found households to not use their closest source for cost and quality reasons. Finally, we explore a synwork of these methodologies to show how the modeling of water access points can give more information on the usage of multiple sources and with this, how SDG Target 6.1 can be better monitored. We find that, albeit a necessary addition, monitoring can not solely rely on data-driven models and should utilise household survey data; as such, current household health surveys should not remain limited in their focus on the primary water source only.

## Results

In the sections below three models are discussed. All models are trained to predict the number of presences for different access types over 1km by 1km gridded map of Uganda. During training, each model learns what combination of socio-economic and geohydrological features can predict whether a cell contains a water point (e.g. presence for a borehole) or that no presence was recorded for that cell (background cells). Two Neural Network (NN) models are presented, the difference between the NN Standard and the NN weighted background is that for the latter, background cells are selected based on population density weights, such that cells with a low population are selected more often as they are considered less likely to contain water access points. The third model is a Maximum Entropy model (MaxEnt), such that our results could be compared to state-of-the art^11^. Some of the predictive model results are also validated on highly detailed household survey we carried out in Bushenyi. The models are described in Section "Water access point presence estimates using WPDx, geohydrological, socio-economic and DHS data".

### Modeling WPDx data

**Figure 3:**
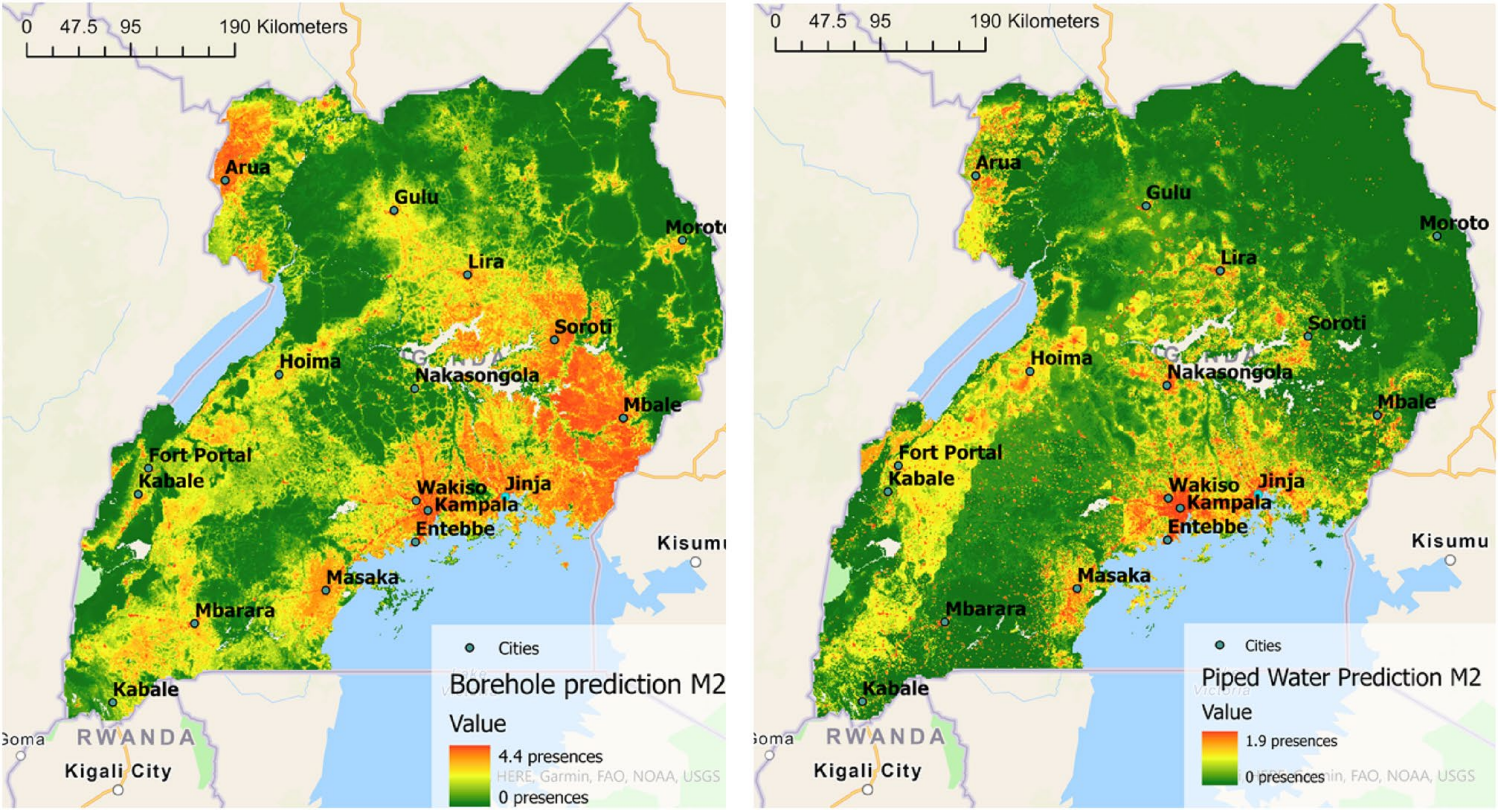
Number of presences across Uganda of Boreholes (left) and Piped Water (right) as predicted by the weighted background neural network model. Initial relative probability model output was scaled using Eq. (11) to represent predicted number of presences. Note that the color-bar for number of presences has a different scale in the left and right figure.

Two examples of the model outputs are shown in Fig. 3; specifically, the predicted number of presences of boreholes and piped water connections in Uganda respectively. The model predicts a higher presence of both access types in cities. However, boreholes are also predicted to be present in rural areas, while piped access is predicted much less in in rural areas.

The model outputs are evaluated by computing the True Positive Rate (TPR) and the False Positive Rate (FPR) of the model predictions on the 30% test set of WPDx data that was kept apart during model training. Table 1 shows that the neural network is capable of assigning almost 80% of the presence cells correctly with a presence, i.e. a relative probability larger than the threshold set at a relative probability of 0.3. Only 30% of the background locations receive a relative probability larger than the threshold. This might seem high at first but this is mitigated by the notion that background locations are expected to contain some presences. When the standard neural network model is compared to MaxEnt, it becomes clear that although the TPRs of MaxEnt are relatively high (Weighted average of 0.85 vs. 0.78 for the standard NN), the FPRs show that MaxEnt has little distinguishing power, predicting a false presence almost half of the time (FPR = 0.46). This shows that, as indicated for species modelling^15^, neural networks perform better in modeling presence only data. The results also show that using only the AUC evaluation criteria presented in other work^11^ hides this distinguishing power; see also Methodology Section 4.5.

**Table 1:** Model performance per access type of the Neural Network (NN) Standard, the NN Weighted Background and the MaxEnt models expressed by True Positive Rate (TPR) and False Positive Rate (FPR).

Access type	N	NN standard		NN weight. backg.		MaxEnt	
		TPR	FPR	TPR	FPR	TPR	FPR
Borehole	36758	0.74	0.34	0.84	0.26	0.89	0.56
Piped water	124	0.94	0.24	1	0.24	0.85	0.23
Prot. well	1632	0.93	0.28	0.94	0.21	0.86	0.25
Prot. spring	28161	0.86	0.30	0.95	0.22	0.87	0.37
Rainw. harv.	18624	0.71	0.24	0.88	0.23	0.74	0.42
Surf. water	535	0.67	.36	0.75	0.25	0.86	0.55
Unp. well	323	0.92	0.26	0.95	0.19	0.86	0.21
Unp. spring	239	0.86	0.26	0.94	0.22	0.81	0.18
	Weighted average	0.78	0.30	0.89	0.24	0.85	0.46
	Average	0.83	0.29	0.91	0.23	0.84	0.35

The threshold for presence (Eqs. (14) and (15)) is set to $\theta =0.3$. Weighted average refers to averaging by the number of datapoints (N).

Introducing the weighted background selection (second column of Table 1) in the neural network model, further improves results. With weighted background selection, presence cells are compared to cells with a small population, as these cells are unlikely to contain many water access points. The results have to be interpreted with some caution as the FPR is computed from the background data which in this case consists mainly of (almost) non populated areas. Compared to the standard background cells, the weighted selected background cells are better distinguishable using feature layers such as population growth and increased nightlight. The TPR however, is computed from presence locations only which are (in the majority of times) populated. Therefore, for the TPR, there is no added advantage compared to the standard model from population related feature layers. This shows that the weighted background model is capable of predicting presence better than the other models. However, because the weighted background model is more ’population’ driven, some areas might be receiving a higher number of predicted presences then they in reality have because for this model the presences are divided over mostly the populated cells.

**Figure 4:**
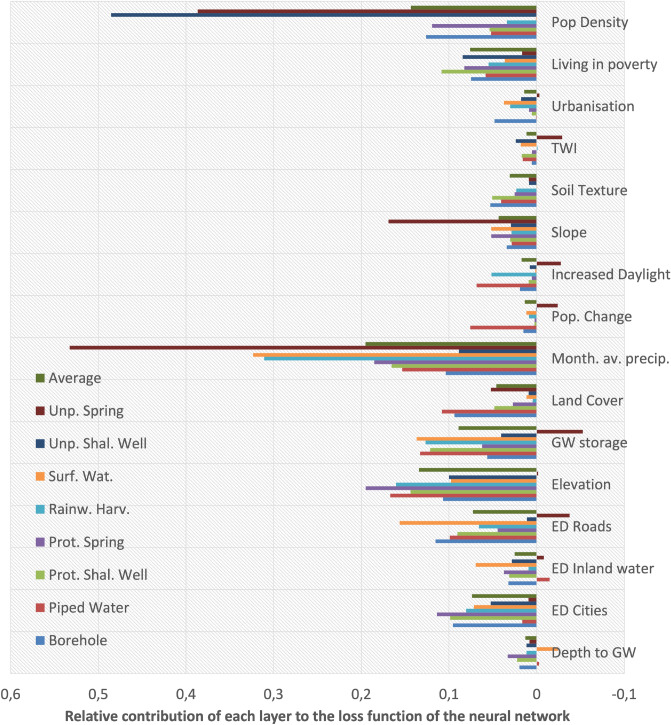
Relative contribution of each predictive feature layer to the loss function (Eq. (7)) for the NN Standard model. The relative contribution is computed from the permutation feature importance, which is the decrease in a model score when a single feature value is randomly taken out. To come to the relative contribution (as displayed in here), these scores are divided by the total loss function of the trained model.

The results in Fig. 4 show that the presence of water access points can be predicted using both feature layers relating to water resource availability, as well as accessibility in terms of road and other infrastructure (ED cities), but also socio-economic indicators. The importance of rainfall for predicting almost all access types is interesting and could indicate that wetter areas have better water access (in Uganda). Furthermore, elevation seems to play a relatively large role in predicting water access, which was also reported as an access predictor for Kenya^11^.

#### Sensitivity of model performance to background data size

In Tables 2 and 3, the output from the neural network model of three access types is shown for different numbers of background points. As expected, if the ratio between the number of background locations and the number of presence locations (N) is low, this results in both high TPRs and FPRs, showing little predictive power with regards to presence. For example, for N/2 background points, the borehole TPR is high with 96% but also 82% of all background cells receive a too high probability. At the same time, a very high ratio in sample size between background points and presence locations results in less false positives, driving down the FPR, while not improving or decreasing the TPR. This can also be seen in the mapped results in Appendix A, showing different spatial distributions of borehole presences in Uganda for different number of background points used. Using too many background points result in overfitted data (only presence locations receive high scores) and too few background points allow for little distinguishing power resulting in very widespread predicted presences. Depending on the access type, an optimum size seems to lie between 2N and 8N background points.

**Table 2:** Sensitivity analysis: the relative number of background points shows a trade-off between performance measures TPR and FPR of neural network model outputs.

# Background points	N/2 TPR (FPR)	2N	4N	8N	20N
Access type					
Borehole (N=36709)	0.96 (0.82)	0.82 (0.47)	0.74 (0.34)	0.42 (0.10)	0.15 (0.02)
Piped water (N=124)	0.95 (0.80)	0.94 (0.29)	0.89 (0.23)	1.0 (0.13)	0.70 (0.05)
Prot. spring (N=28155)	0.96 (0.73)	0.90 (0.40)	0.82 (0.25)	0.46 (0.09)	0.37 (0.03)

Here *N* represents the number of presence locations (e.g. number of WPDx borehole points) used in training.

**Table 3:** Sensitivity analyses to show impact of the number of background locations on the AUC.

Access type	AUC (N/2)	AUC (2N)	AUC (4N)	AUC (8N)	AUC (20N)
Borehole	0.84	0.84	0.84	0.83	0.84
Piped water	0.90	0.99	0.94	0.99	0.98
Prot. spring	0.92	0.93	0.93	0.94	0.93

Contradictory to the TPR and FPR, AUC is not sensitive to the number of background locations used; here N represents the number of presence locations in the training data (e.g. number of WPDx borehole points).

In Table 3, it can be seen that for different numbers of background points, despite changing TPR and FPR, the AUC remains the same. This is misleading, especially when looking at the example output in Appendix A, where it can be seen that the distribution of predicted presence of the access types differs significantly for different number of background points. As explained in Section "Validating model performance", the unchanging AUC can be attributed to the fact that AUC weighs the TPR and FPR equally and this allows the scores to make up for each other resulting in constant (high) AUCs. Naturally, to increase the TPR, changing the threshold $\theta$ for presence to lower values when the TPR becomes too low would be an option. However, as the relative probability is scaled eventually using the total number of presences and the threshold does not influence the distribution this is not done here. Besides, keeping the threshold constant for the different access types allows for better comparison of the models predictive performance.

### Survey: water access in Bushenyi and associated health risks

Table 4 shows the share of the Bushenyi population that reported to regularly use the listed sources, which demonstrates that households in Bushenyi make use of multiple sources for their daily water supply. More specifically, households reported to use an average of 1.9 sources for their general water supply. From the respondents, 66 out of 517 (13%) reported that their closest water source (CWS) is not their primary water source (PWS). This is a decline from 2018 when this was the case for one in four households^3^. This decline is probably a result of the recent increase in piped connections in the area: 50% of the users with piped water as PWS, have only been connected in the last three years. The reason for not using the closest water source is mostly the price (43 out of 66), unaffordability of piped water seems to have caused some users to resort to free sources. Consequentially, the higher income and better-educated groups have significantly more access to piped water than the poorest households (for more details, see Appendix B). The travel time to the CWS is 4.6 minutes on average, which is significantly less then the 20 min average travel time to and from the PWS, with Wilcoxon’s rank-sum: w(128)=7.44, p$<<$0.0001. This indicates that people are willing to travel an average of 15 minutes longer for their daily water supply to avoid (mostly) costs. When asked to score the reliability of their CWS and PWS between one and ten, respondents gave the PWS an average of 8.3 and the CWS a 7.5 (w(128)=2.07, p=0.04). With regards to both physical and health safety and cleanliness, the PWS and CWS received very similar scores (all around 8).

**Table 4:** Bushenyi survey results showing the share of households that said they have access to and do regularly use the listed sources.

Access type	Share of population with access
Piped	0.56
Borehole	0.03
Shallow wells	0.28
Springs	0.43
Surface water	0.09
Rainwater	0.51
Other	0.04

From the respondents, 155 (30%) said they do not drink from their CWS. This time, safety (84), cost (38) and bad taste (24) were the predominant reasons. More specifically, for households with piped access, 85 (29%) reported not to use the closest water source as drinking water source; deeming it unsafe (47), expensive (22) and having bad taste (20) as the most listed reasons. The enumerator team also recalled that especially people that have only recently been connected to the piped network, still are traditionally very accustomed to get their water from springs (also reported for Bushenyi before^3^). The enumerator team had also noted that people in more urbanised areas of Bushenyi are more comfortable with drinking piped water as they have had access for a longer time and also because it is often the only source available nearby. The survey results indeed showed that the urban areas have more piped connection and that especially the hilly rural areas in the North-East of Ishaka have no piped water access since they are hard to connect.

Even though almost all respondents stated they treat their water before drinking, 154 households admitted drinking untreated water at least once in the past four weeks. Out of 517, 23 households (4%) reported having at least one member suffering from diarrhea in the previous 30 days. Similarly, 97 (19%) suffered from respiratory illnesses. The results show that drinking from an unprotected source does not seem to (significantly) increase the risk to contract these diseases in Bushenyi (similar to what is reported earlier^18^). However, drinking untreated water, doubles the risk of diarrhea since 8% of households that reported drinking untreated water at least once had suffered from diarrhea; comparing the population proportions using a z-test gives: z=2.0, p=.02. It is also associated with a 10% increase in respiratory illnesses reported in the survey (z=2.7, p=.004).

### Model performance for Bushenyi

Table 5 shows how capable the model is of distinguishing the Bushenyi cells in which usage of the listed access type was reported in the survey from cells in which this was not reported. Here it is expected that the models predict many borehole presences in cells comprising households that report to use boreholes, and little borehole presences for cells in which this was not reported. In total, 63 cells were surveyed, subsequently, households located in the same cell, receive the same model prediction per access type. Next, the model predictions of the households that did report using a borehole are compared to the model predictions of the households not using a borehole, and likewise for the other access types.

**Table 5:** Comparison of predicted presences averaged over Bushenyi grid cells for the standard Neural Network Model with survey data from Bushenyi households indicating using or not using the listed access type.

Acc. type	HHs using	Model predicted presences	HHs not using	Model predicted presences	Statistics	
					w	p
Piped	288	2.53	229	1.65	4.72	0.00
Rainwater	263	0.15	254	0.14	2.61	0.01
Borehole	15	1.00	502	1.06	− 0.89	0.37
Shallow wells	143	1.40	374	1.18	3.42	0.00
Springs	224	1.08	293	1.15	− 1.85	0.06
Surface water	44	0.88	473	0.99	− 1.25	0.21

Note that the model scores are from a gridded 1 sq. km. output, where all households in that cell get assigned the same prediction. In total there are 63 different model cells surveyed, resulting per access type in a total of 63 possible model outputs of the households. The symbols ‘w’ and ‘p’ stand for Wilcoxon rank-sum score and significance.

In Table 5 it can be seen that the models for piped water access, rainwater harvesting and shallow wells perform as expected, assigning significantly higher predictions to cells where these access types are used. It indicates that in Bushenyi features are distinguishable enough for the model to predict the (non)-presence of these access types. It is noticeable that these three access types are used by a large share of the households too, probably providing large enough samples to make a significant comparison here compared to other access types.

For boreholes and surface water access, the model seems to underperform but it is not possible to draw statistical conclusions. For springs however, the model behaves unexpectedly, assigning significantly lower predictions to cells in which households report making use of springs. This could mean the model is off, for instance not capturing the fact that people might travel outside their cell to a spring. This latter would make sense as earlier work^3^, the survey results and local team reported a preference for springs for cultural traditional, taste and cost reasons.

In Fig. 4, it is shown that poverty, precipitation and elevation are important drivers of water access on the nationwide scale. Likewise in Bushenyi, we find that the North East region has a higher altitude and a larger share of households with lower incomes and at the same time, worse water access i.e. less protected water sources and less piped connections. The poverty aspect is shown in more detail in Appendix B as well, where it is shown that the households with lower incomes have worse and unsafe water access more often.

## Discussion

The sections below provide a critical assessment of the results by placing them in perspective to each other and to the state of the art from literature. By comparing the results from the machine learning models at nationwide scale with the locally executed survey, we reflect on how predictive features scale and how spatial heterogeneity might impact results.

### Modeling water access levels nationwide

In this work, we extend the Max-Ent modeling technique proposed in the work by Phillips et al.^19^, with more general neural network models. With the neural networks, improvements were made to model the distribution of water access points across Uganda for eight different water access types. The results show that it is possible to model the water access point distribution over the country, also into areas that were not covered by WPDx. The model was tested on 30% of the WPDx data that was kept apart during training. Most of the model setups perform well on the predefined evaluative parameters and assign (much) higher scores to water point presence locations than background locations, for all access types. The sensitivity analysis showed that the number of background points can significantly influence the model output. However, despite being often used for evaluation in this setting, the AUC measure is insensitive to this. This justifies a choice for the True Positive Rate (TPR) and False Positive Rate (FPR) for evaluation in this setting.

Our results in Section  "Modeling WPDx data" show that the neural networks used, outperform the MaxEnt model in modeling presence only water point data in Uganda. This is in line with the prior assumption that neural network models are capable of capturing more complex and non-linear transformations and combinations of access determining geographic and socio-economic features than MaxEnt^15^. Therefore, the expectation is that more complex functions than the logistic parameterisation of MaxEnt are required to best model water access.

The introduction of a weighted background selection based on population density, further improves the neural network results, achieving a TPR of almost 90% and an FPR of only 24%. The introduction of a method for scaling the relative probability output to an absolute number of presences, gives more information on the co-existence of water access types and allows for better multi-source water use monitoring. It is therefore an improvement on earlier water access point modeling^11^ and potentially for species presence only modeling in general^12^.

Precipitation, elevation, population density, poverty and groundwater storage were found to be important indicators for the (non)presence of water access points. This is in line with the prior assumption that water access is related to both socio-economic conditions as well as natural water availability and (probably) terrain suitability. As water access type modeling to our knowledge has little precedent, we have to analyse these results in detail to place them in perspective. For instance, the relative contribution of the feature layers to the loss function showed that, although slope is not an important predictor for most access types, it is for unprotected springs (Fig. 4). This coincides with earlier work that showed that the terrain slope is the most important factor for unprotected spring presence in Iran^20^. Other work found that rainfall and elevation were important predictors for both unprotected wells and surface water access^11^ in Kenya, which we see coming back in our results for Uganda as well. Results from the same work, also place a high weight on urbanisation^11^, which is less dominantly present in our results. This can be explained by the fact that there, the population density was not included as a feature layer, which is assigned high weights in our results.

The reported TPR and FPR show that our models mostly perform well as indicator for the presence of water infrastructure on the national scale. Using the Bushenyi survey for a more detailed evaluation of the weighted background model’s performance, it was shown that, also on this detailed survey scale, the models are still able to pick up some water access important features such as elevation and poverty level. The respective models assign significantly larger scores to cells with households that report piped water use ($p<0.005$), rainwater harvesting ($p=0.06$) and shallow wells ($p<0.005$) respectively when compared to cells that do not report using these access types. However, for springs the model is underperforming, assigning lower scores to cells with households using springs ($p=0.05$). This can potentially be attributed to the local preference for springs for taste, cost and traditional reasons, as reported before in Bushenyi^3^. This shows that the model outputs can be good indicators for the presence of water infrastructure but that the actual utilisation of a source is, for some sources, harder to predict. The latter is a result of complex local dynamics, e.g. neighbours using completely different access types and people not (only) using their closest source but a multitude of sources.

### Most households in Bushenyi use more than one water source

The results show that in Bushenyi, households make use of an average of two water sources on a regular basis and over one hundred households (out of 517) reported using three or more. Our results indicate that out of 293 households with close-by piped access, 85 (29%) hardly make use of it for cost or quality reasons. This exposes some shortcomings of JMP’s reporting on safe water access: JMP considers people with a piped water access point on premises, to have improved water access (i.e. achieve SDG target 6.1)^6^.

Furthermore, similar to earlier work^18^, our results indicate that having no access to water from protected sources does not imply an increased risk of diarrhea and respiratory illnesses. Only in cases where it was reported that “water is (sometimes) not treated before drinking”, we found that the household had a significantly higher reporting of catching these illnesses (up to double the risk, see Section "Survey: water access in Bushenyi and associated health risks"). In Bushenyi, almost one in three households reported “not treating their water before drinking it” at least once in the past four weeks. The WHO did find an increased risk of diarrhea for unprotected source users, but likewise report that proper and constant water treatment has a larger positive effect for prevention^21^ than drinking from protected sources. All of this questions the usefulness of making an absolute distinction between protected and unprotected sources as our results show that this is not related to the mentioned diseases. This critique can be extended by the notion that surveys on water access and consumption focus too much on the primary (drinking) water source (also reported before^4^). In Bushenyi we saw that many people make use of a variety of sources both protected and unprotected. These people will, although not daily but every now and then, drink untreated water or water from an unprotected source. The implications of this are such that a focus on the primary (drinking) water source in surveys combined with the focus on the protected/unprotected distinction, can cause an overestimation of the population that has permanent safe water access. On the other side, however, the usage of multiple sources can bring resilience to droughts or other failures of water sources^4^. Therefore, we argue that a larger emphasis must be placed on always treating water and not to let unprotected versus protected water access be leading when assessing the level or safety of water access in developing countries. Furthermore, we suggest that health surveys should better map the variety of sources used by households as this can have both positive and negative effects with regards to the safety and availability of water access.

Since our proposed models make predictions per access type, this does allow for the modelling of multiple of access types per grid cell. In that way, the presence and the (variety) of type(s) of water access points can be indicated, providing a possible monitoring alternative or addition to health surveys. From the model alone, it is however not always possible to be certain that these access points are used as we saw with the local preference for springs and households reporting not using the piped connections in Bushenyi. Ideally, locally obtained knowledge on water price but also cultural preferences for particular sources would be included. This could be obtained by expanding some of the DHS questions with questions on the variety of and preference for water sources used as we did for Bushenyi.

Another noteworthy result is the low number of rainwater harvesting systems predicted by the model (0.15 systems per cell, see Table  5) compared to the reported usage in Bushenyi (half of the households have access). A possible explanation is that the total number of expected presences, which is used to scale model output, could be wrong (as explained in Section "Estimating total number of water access points per grid cell"). The estimated total number of rainwater harvesting system presences is based on DHS numbers and the general critique on such water access monitoring surveys is that they happen during dry seasons^4^, not capturing the complete rainwater harvesting usage. For this work executed survey campaign took place at the start of the wet season when rainwater harvesting is expected to be more predominant.

### Limitations

The use of packaged and bottled was not modeled because WPDx did only contain one data point for Uganda. However, there is anecdotal evidence for packaged water to be used throughout Africa for drinking purposes. A second limitation lies in the used datasets. In the used WPDx data, 79% was functioning, 18% was not and for 2.5% it was unknown. Modeling only the functioning sources would be an option but because some sources might have been fixed (or broke down) since registering, it was decided to model all, allowing for a larger data-set. This does add a limitation as one can not be fully certain that modeled access points are functioning. Further researching the frequency of breakdowns of water access points, potentially using more WPDx information, is advised. Thirdly, the influence of the number of background points used for the classifying models should be researched further. The sensitivity analysis showed that changing the number of background points changes the distribution, where too many background points lead to overfitted results with low predictive power, and too little background points lead to distributions that can hardly distinguish areas with from without presence. This also connects to sampling bias correction methods. There is not very clear guidance on what bias correction method to apply for species modeling^22^. It was shown that the ability of methods to correct the initial sampling bias varied greatly depending on bias type, bias intensity and species^22^. For the context of WPDx modeling it is unclear what would exactly be the bias type as some access types are surveyed rather cross-country and other access types seem very localized. A logical distinctor for the bias is the population presence as where people live we are more likely to find water access. This is applied as a bias corrector in the weighted background modeling method. For the other two models and for fairness of comparison, no bias correction is applied which is a limitation of this study. Furthermore, there might be other models that can outperform the models presented in this manuscript. The applied modeling methods were limited to the MaxEnt and the MLP models, but there are many others out there. Further research could focus on which type of model is best placed to model water access points. Lastly, we suggest to research further the number of people that are served per access type. Our approximation from Section 4.3 is under two important assumptions namely that the access points are all serving at their limit and that the WPDx data from cells that were surveyed for WPDx is complete, containing all water access points of the cell. Already in the Bushenyi data analysis it was found that the models grossly underestimate the usage of rainfall harvesting. This is a result of the low reported usage in the DHS data. If the number of presences turns out to be very different than approximated in this work, the predicted number of presences accross Uganda would be different. However, the relative probability distribution of access types would remain the same, making it mostly a scaling issue. It would be interesting to validate the number of users per source for our research but also because there is very little information on this in literature. For the survey, the most important limitation lies in the fact that the campaign took place during one of the two Ugandan wet seasons. To get a further understanding of the water consumption and peoples water behaviour, we recommend to perform a similar survey in a dry season and to look how that influences water consumption, travel time and water access in general. For instance, almost half of the households using rain water harvesting as primary water source reported that this is only the case in the wet season.

### SDG 6 monitoring improvements

This work has shown that geographic inequalities in access to improved water can be modelled through the use of sample WPDx water point data and DHS data; highlighting at high spatial resolution on which areas in a country are likely to have good water access and which do not. This can be used by governments, water companies and NGO’s to prioritise areas with poor access for water access campaigns and can help with better SDG 6 monitoring in general. In addition, the feature layers can also give an indication as to why an area has poor access; whether it is a resource problem or whether a socioeconomic determinant is more relevant? The model results showed for instance that water access is generally high in urban areas compared to rural areas as the model expects larger number of water access points in urban areas. Geographically, the North of Uganda gets less rain than the South and also has lower groundwater storage, this too results in less predicted water access points, mainly because the water resource availability is less (as also indicated in previous studies of water stress in Uganda^23^). Elevation was also found to be an important indicator; it is possible that not the elevation itself but more that the mountainous areas having steep difficult terrain and deeper groundwater makes them areas suited for most water access types, including piped water infrastructure. For the surveyed area, we also found that especially the higher, hilly more rural areas of Bushenyi had worse access than the urbanised lower areas, coinciding with the model results.

Still, as the survey in Bushenyi has shown, the actual presence of a source, does not always mean that it is used. Besides that, training data for the models remains necessary and the relation between the training data and the feature layers may also change as new water technology or customs change. Therefore it is no option to rely solely on data driven WPDx models alone for SDG 6 monitoring. (Health) surveys remain necessary to get a complete picture on water use but should be extended with questions on multiple sources that are used by the household. Without adding this nuance, the number of people that have permanently safe and affordable access to drinking water is at risk of being overestimated^4^.

## Methods

**Figure 5:**
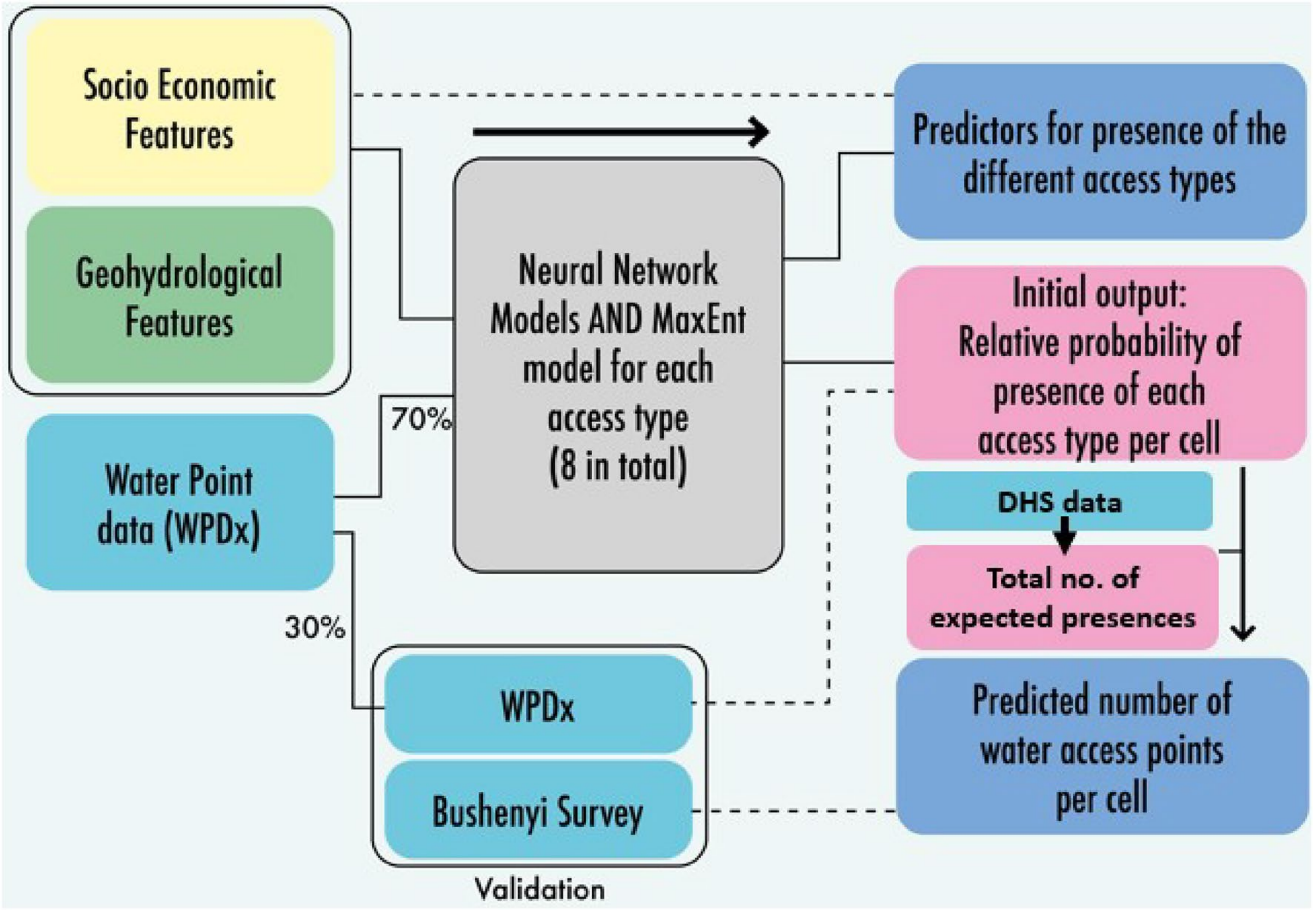
Modelling framework where neural network classifying models and a MaxEnt model are trained to predict the presence of water points (of 8 different access types) using predictive socio-economic and geohydrological feature data. The initial output of such models is the relative probability of presence per grid cell e.g. a borehole. At that stage (pink box) the model output is validated using the remaining 30% of WPDx data. By using an estimation of the total number of expected presences, the initial output is scaled to the total number of water access points per cell. This spatial output is compared to our own survey data at household level water access in Bushenyi.

In this research, a dual methodology is applied consisting of both a modeling and a surveying part. As per the models, the distribution of water access points across Uganda is modeled using advanced biological species machine learning modeling techniques. This is done per access type such that there is a model for piped water, one for boreholes and the other access types as indicated in Table 6. The output is a gridded map with the predicted number of water access points for each access type. The applied machine learning models make predictions of water access point presence, using several geohydrological and socio-economic feature data-sets. In parallel to the development of these models, a survey was executed in Bushenyi, explicitly researching the usage of multiple sources and the local dynamics of water consumption but also to validate model results. An overview of the model setup and the validation processes can be seen in Fig. 5.

### Study area and grid cells

The study area of this research is Uganda. The East African country with 44 million inhabitants of which 78 % have access to safe drinking water^5^. Despite that, almost half the population still travels over half an hour to their primary water source^5^. The survey campaign took place in Bushenyi, which is located in the South West of Uganda, close to Lake Edward. The Bushenyi area has experienced and is undergoing rapid urbanisation shown by the steady population growth of 6 % for the past 5 years. Bushenyi town is the administrative centre of the Bushenyi district^24^ that is now housing 250 000 people (according to https://bushenyi.go.ug/district/statistics). As the town is growing and urbanizing more, many households switch to a piped water system that is provided and maintained by the National Water and Sewerage Corporation (NWSC). Furthermore, Bushenyi is an interesting research location because of the large part of the population living ‘in between’ environments combining both urban and agricultural characteristics^3^. Researching such locations is relevant as urbanization is expected to continue and especially midsized and small cities are often overlooked in research^25^. An interesting conclusion from an earlier survey in Bushenyi was that one in four households in Bushenyi do not make use of their closest water source^3^. This is already an indication that the presence of a water source not always means that it is used in reality.

Since most of the input feature data and all of the output of the model are described at the scale of gridded cells, we define them here first. If we were to place a rectangle on the map of Uganda and divide that rectangle in squares of 1 km$^2$ each, each of these squares represent a cell. In total this results in 686 x 652 cells. Of course some of these cells lie outside the boundaries of Uganda, which are automatically excluded from the set of cells used for model training and validation. Eventually, predictions will be made in terms of water access points per cell (e.g. 5 boreholes in cell x).

### Data

#### Water point data

**Figure 6:**
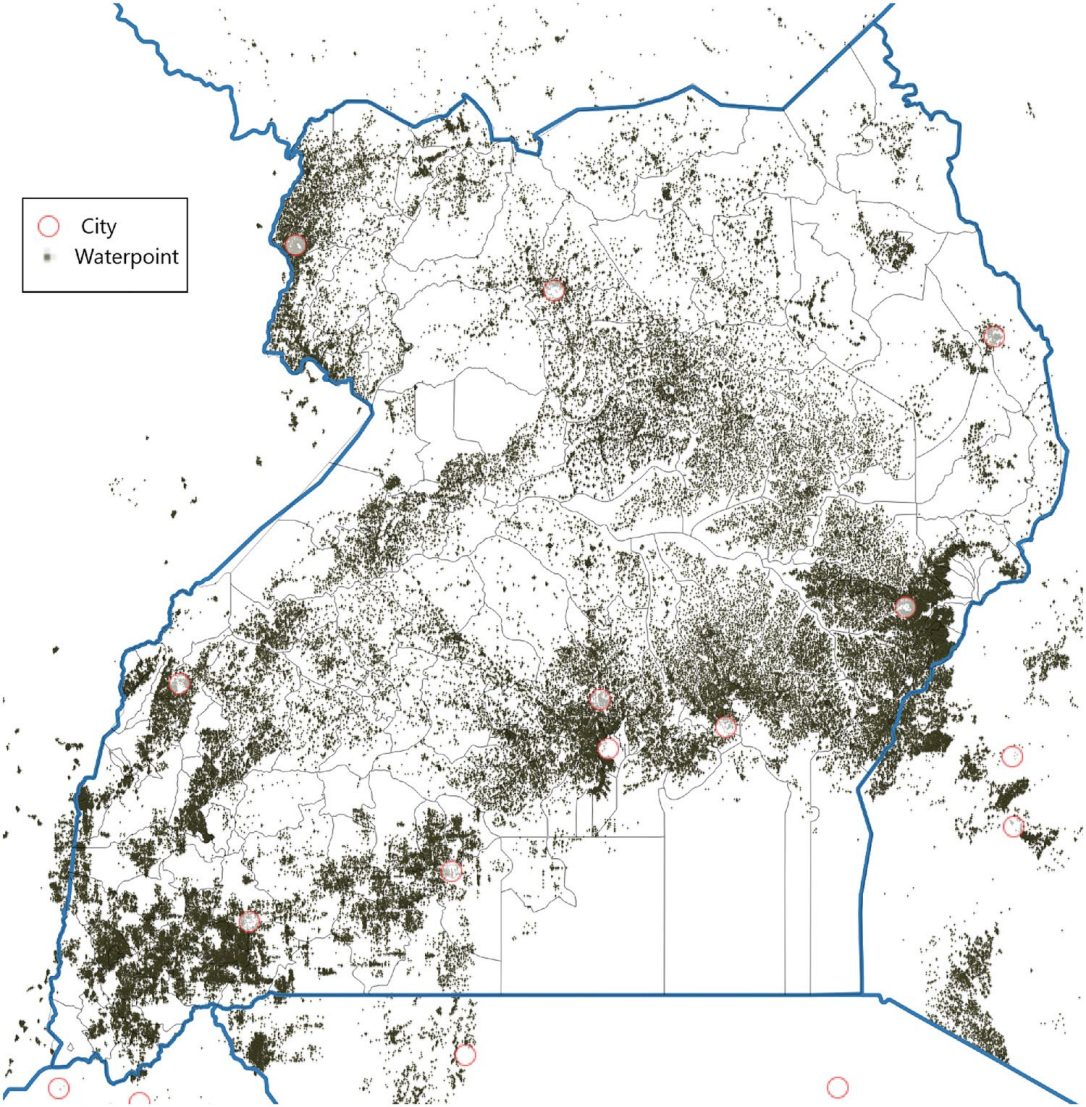
Water Point Distribution across Uganda. Figure made with ArcGis Pro.

All available Ugandan water point data was extracted from the Water Point Data exchange (WPDx, https://www.waterpointdata.org/) in September 2021. A water point data consists of the geographical coordinates and the type of water access type present at that location. The distribution of the WPDx data across Uganda can be seen in Fig. 6. Note that this is the data from all the considered access types (boreholes, wells, etc.) and that for each individual access type the nationwide coverage is not nearly as high (see Fig. 2). The different water access types and their number of presences in Uganda are shown in Table 6. This is not exactly the true map of water access types used in Uganda, it only represents which access types were registered on the WPDx portal. In total there are 121 331 WPDx points for Uganda, which averages to 1 access point per $5 \text { Km}^2$ or roughly 1 access point per 230 inhabitants. This was above average when compared to other countries on WPDx portal, also motivating the use of Uganda as a case study.

**Table 6:** Water access types and their corresponding number of presence data in the WPDx database for Uganda.

Access type	Number of WPDx points	Improved access type	Modeled?
Borehole	36758	Yes	Yes
Packaged water	1	Yes	No
Piped water	124	Yes	Yes
Protected shallow Well	1632	Yes	Yes
Protected spring	28161	Yes	Yes
Rainwater harvesting	18624	Yes	Yes
Sand or sub-surface dam	1	Yes	No
Surface water	535	No	Yes
Undefined shallow well	18260	Unknown	No
Undefined spring	18	Unknown	No
Unprotected shallow well	323	No	Yes
Unprotected spring	239	No	Yes

The table also indicates whether these are considered as improved/unimproved water sources by JMP^6^, and whether the sources are considered in our models.

Previous work has considered the extrapolation of Kenyan water point data for Surface Water and Unprotected Dug Wells using MaxEnt models^11^. In this research we consider all water sources displayed in Table 6, excluding the sand surface dam, packaged water and the two unknown facility sources. The first two are excluded because the data sets are too small and the latter two because it is important to predict whether access is deemed protected or unprotected (improved/unimproved), as this gives information on the hygienic safety of the source. In this manuscript, we also consider the more general nonlinear NN models and a wider set of resource and socio-economic features, including data from household health surveys, to estimate the density of access types per unit area, in addition to probabilities of presences.

#### Presence only data

**Figure 7:**
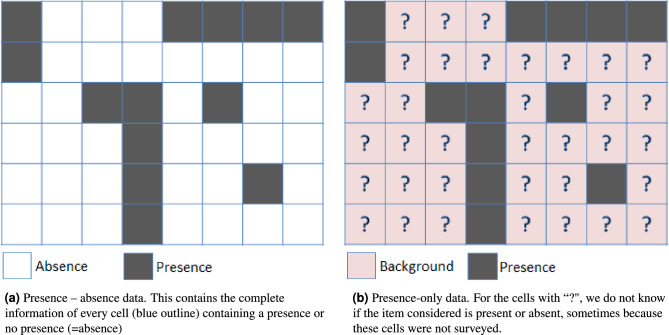
The difference between presence-absence data (left) and presence-only data (right) is that for presence absence we have full information on both presence and absence of surveyed cells while with presence only data we have only full information on the cells containing presences while for the other cells presence is unknown. Figure inspired by Ref.^26^.

In the biological species modeling field, the objective is usually to find species geographic distributions from a set of georeferenced species observation data (i.e. we want to find a model that can predict a species (potential) habitat using environmental variables that likely determine suitability for the species). Biological species modelers usually refer to the mentioned observations of the species as point data. Such data is typically subdivided in two categories: presence-only and presence-absence^13^. If we take the species modeling example of a daisy flower: for presence absence data (Fig. 7a), a (random) selection of cells in the study area is researched and both the cells with daisy flowers (presence) as well as the cells without daisy flowers (absence) are registered as such. This allows for a strong comparison between the environmental features enabling (presence) or disabling the presence (absence) of daisy flowers. Presence only species data is, for example, mostly a result of decentralised event based methods of data collection that are not systematic or organised, where it is only possible to ascertain presence of a species via observation events at a (limited) sample of locations spatially. In many cases, this may also be due to logistical or resource limitations that make it prohibitively expensive to survey the whole geographic space of interest or to ascertain absence^13^. For presence only data, we only know that some cells with presence observations do contain daisy flowers (presence); for the other group of ’background’ cells (pink in Fig. 7b), it is unknown whether they contain daisy flowers or not. Still, if the sample is large enough, the presence only data can be compared to (randomly) selected background cells which then are often referred to as pseudo-absence cells. This presence-only modeling technique is under the assumption that the species (daisy flowers) is not prevalent in the entire study area. In the following, we use “presence only” water point data from WPDx, and multiple geospatial features to model water access and identify its determinants.

#### Feature data

Maps were selected that hold information on environmental, geo-hydrological, socio-economic and technological features that potentially indicate a cells suitability for (one or more of the different) water access types. All the used feature layers and their sources can be seen in Appendix C and are summarised in Table 7. As machine learning models are used, we let the models select the most suitable feature layers from the lot instead of deciding this upfront. The criteria for choosing these feature layers are in Table 7. The layers are visualised in Appendix C.

**Table 7:** The feature layers used as water access point predictors in the models, the reason to include them and their data type. For a more detailed overview including sources, see Appendix C.

Feature data	Reason to include	Data type
Euclidean distance to inland water	The proximity of water relates to water access.	Numerical
Euclidean distance to residential areas	Where people are present, there is often water.	Numerical
Euclidean distance to cities	Cities are expected to have better water access.	Numerical
Groundwater storage	Presence of groundwater is needed for some access types.	Categorical
Slope	Can influence the suitability of terrain for water access types.	Numerical
Soil texture	Can influence the suitability of the terrain for water access.	Categorical
Urbanisation	Urbanised and less urbanised areas allow for different water access types.	Categorical
Topographical Wetness Index	Can influence water proximity.	Numerical
Population density	Dense and less densely populated areas allow for different water access types, besides people tend to live where water is present.	Numerical
Land cover	Different land cover types could allow for different water access types.	Categorical
Monthly average precipitation	Essential for rainwater harvesting but could also influence other sources.	Numerical
Poverty	Richer households tend to have better water access.	Numerical
Depth to groundwater	Presence of groundwater is needed for some access types.	Categorical
Groundwater productivity	Presence of groundwater is needed for some access types.	Categorical
Population change 1990-2014	Areas that urbanized very quickly have sometimes worse water access.	Numerical
Increased nightlight 1992-2013	Same as above	Numerical

All maps are resampled to a 1 $km^2$ (= 1 cell) resolution using ArcGis Pro. Most maps have that or a higher resolution. During resampling, for numerical data bilinear interpolation is applied and for categorical data nearest neighbour resampling. All the used feature datasets are freely available from the listed sources making this method accessible for all and reproducible.

As suggested in other work^12^, to reduce colinearity, the feature layers are checked for correlation. This is done using Pearson’s R for numerical to numerical data, Cramer’s V for categorical to categorical data and $\eta ^2$ for numerical and categorical data. It was found that groundwater productivity and groundwater storage were correlated (Cramer’s V = 0.68) and secondly the euclidean distance to roads and to residential areas was correlated as well (R>0.7). The euclidean distance to roads as well as the groundwater productivity layer were therefore removed from the set of feature layers.

### Water access point presence estimates using WPDx, geohydrological, socio-economic and DHS data

The WPDx data is incomplete for most access types, i.e. it does not cover the entire nation for many countries including Uganda. Therefore, this research presents models that predict the presence of these access types over the entire geography of the nation. Machine learning models will be trained and used to make predictions for all grid cells. During training, the model uses feature layers to find distinguishing information between presence locations and background locations (e.g. high rainfall together with higher income may be predictor features for rainwater harvesting). We therefore use feature data sets that are available for grid cells nationwide to make nationwide predictions of the presence of the different access types. In the following, we first introduce the mathematical formulations of prediction with incomplete presence only data and then discuss three models with different setups.

Mathematically, our objective is to estimate an unknown probability distribution $\pi (x):=P(x\vert y=1)$ that assigns to each point *x* (i.e. a cell or pixel *x* in our study area) a probability that (a source type) *y* is present at *x* given that it is also present in the study area *X*; we assume, for *X* defined as the set of all cells considered as the study area, that $\pi$ sums up to 1 for the finite set *X*^27^. A more intuitive but equivalent formulation considers presence probabilities (for each source type) *y* conditional on the probability distributions of predictive variables (or features); i.e. by means of Bayes’ theorem the following relation holds^27^:

1 $$\begin{aligned} P (y=1 \vert z ( x )) =\frac{P(y=1)\cdot f_1(z(x)\vert y=1)}{f(z(x))}, \end{aligned}$$

where *y* is an indicator variable for the presence of a source type, $X_{1}=\{x: x \text { is a cell where the source type is present, i.e. where } y=1 \}$, *f*(*z*) and $f_{1}( z )$, respectively, represent the probability density functions of the features over the study area *X* and over the presence locations $X_1$, respectively. Therefore, we can estimate the presence probability for a source from the prevalence and the ratio $f_1(z)/f(z).$ However, without reliable presence-absence data (see Section "Presence only data"), the probability of a randomly selected cell to contain a presence ($P(y=1)$), is unknown^28^. When no presence-absence data is available, species modelers often model presence-background data. For such models, the features at presence locations are compared to the features at randomly selected background locations in *X*. These background locations are treated as absence locations (y=0) but in fact this is uncertain as $f_1(z)$ is unknown and thus some background locations are in fact unconfirmed presence locations. This method does however allow for estimating the ratio:

2 $$\begin{aligned} p^* = \frac{f_1(z \vert y=1 )}{f(z)} , \end{aligned}$$

which will be referred to as the relative probability from here on. Therefore, the probability of presence can be derived by multiplying $p^*$ with a constant that estimates the prevalence. In estimating with such models, we assume the prevalence to be 0.5 unless we have more knowledge for a better estimate^27^. In the following, we describe how the Max-Ent and two Multi-Layer Perceptron (MLP) classifier approaches can be used to estimate this ratio of distributions.

#### Max-Ent modelling

One of the best performing and most popular models used for presence-only species distribution modeling is the Maximum Entropy (MaxEnt) model^13,29^. The MaxEnt model has a simple and precise mathematical formulation, and a number of aspects that make it well-suited for presence-only data^19^. MaxEnt parameterises the distribution $f_1(\cdot )$ as a function of $f(\cdot )$ based on the assumption that the distributions on $X_1$ and *X* are similar and differentiated by exponential functions of the features:

3 $$\begin{aligned} f_{1} (z) = f(z) e^{\alpha +\beta ^Th(z)}, \end{aligned}$$

where $h(\cdot )\in \mathbb {R}^n$ contains feature functions that are selected from as a set of basis functions, and $\beta \in \mathbb {R}^n$ are a set of coefficients to be identified, and $\alpha$ a normalising constant that insures the distributions integrate to 1^27^. The distribution *f*(*z*) is estimated from the set of feature data over the study area *X* and the method constrains the mean of $f_1(z)$ to be the same as (or very close to) its mean across the observed set (i.e. presence location data we have).

The Max-Ent model then optimises the objective function^27^:

4 $$\begin{aligned}{}&\max _{\alpha ,\beta } \quad {\frac{1}{m}} \sum _{i=1}^{m} \ln \left(f(z^{(i)})e^{\alpha +\beta ^Th(z^{(i)})}\right)-\sum _{j=1}^{n} \lambda _j \vert \beta _j \vert , \\&\quad \text {s.t.} \int _{X} f_1(z)dz=1,\\&\quad \int _{X} f_1(z)dz^k\approx \int _{X_o} f_1(z)dz^k\ ,\\ \end{aligned}$$

where $z^{(i)}$ is the features vector at location *i* of presence samples, $dz^k$ stands for the differential of the k-th feature in *z* and $X_o$ is the set of *m* locations with presence observation data.

In Eq. (4), the maximisation of this log likelihood term is equivalent to minimising the relative entropy of the predicted distribution ($f_1$) with respect to the prior, while the constraints enforce that the distribution at presence locations $X_0$ has a similar mean as background locations *X*^12^. The regularisation term on the other hand increases model generalisability by trading off model complexity (i.e. having many combinations of features *h*(*z*)) with model fit on the observation set. As in many NN applications^16^, the weights terms $\lambda$ can be optimised to effectively select the most predictive features.

During training, the model tries to find the values (or mathematical transformations of these values) of the feature layers (from Table 7) that are positively associated with presence locations and negatively with background locations. For MaxEnt, these mathematical transformations in $h(\cdot )$ are limited to linear, bilinear, quadratic, hinge, threshold and categorical functions^27^. As a result, the only possible pairwise effect of feature layers to predict a presence is a product function, while the (non)presence is expected to be related with more complex relationships (i.e. combinations of feature layers)^15^.

#### Multi-layer perceptron (MLP) model

In species modeling settings, it has been shown in other work^15^ that NNs can outperform MaxEnt despite MaxEnt’s generally good performance. Because NNs are known to find solutions for very complex non-linear problems^15,30^, e.g. in other work^16^, it was decided to consider several deep NN models as well. Building on the MaxEnt technique, such models were expected to outperform MaxEnt as NNs allow for the modeling of more general and complex nonlinear behaviour than the parameterisation in Eq. (3); as general function approximators with more complex combinations of feature layers are expected to more accurately predict presences. As we are dealing with a classification problem, i.e. determining whether a cell is a presence or a background location, a logical choice is to use a multi-layer perceptron (MLP), one of the most widely used classifying models.

**Figure 8:**
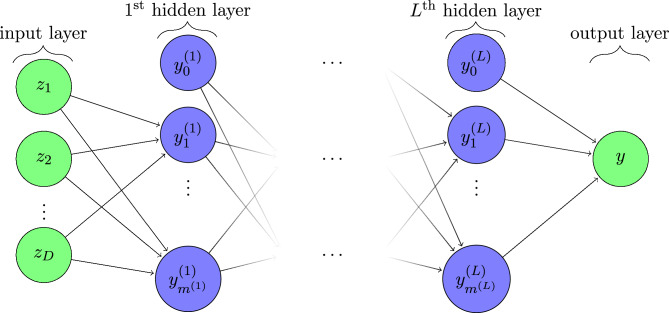
Network graph for a multi-layer perceptron with *L* hidden layers and *D* input features. $[z_1,z_2,...,z_D]$ represent the *D* input features from Table 7, ($y^{(1)},\ldots$, $y^{(L)}$) are the vectors of values in the *L* hidden layers and output layer is (*y*). The hidden layer neurons execute a weighted sum of the outputs from the previous layer plus a bias term, which then passes through a non-linear activation function (Eq. 5)^31^. The $l^{\text {th}}$ hidden layer contains $m^{(l)}$ neurons. Figure adapted from^32^.

To explain the model in more detail, we use the typical MLP classifier visualised in Fig. 8, often used to depict deep neural networks^16^. With the predictor features from Table 7 as the *D* inputs, the model is trained to predict the probabilities of presence $P(y=1)$ in each cell. In each hidden layer of the model, a neuron applies a weighted linear summation to the outputs of the previous layer; for example, $y_1^{(1)} = g(w_{1,1}z_1+w_{1,2}z_2+...+w_{1,D}z_m+b_1) := g(W_1^Tz+b_1),$ where the non-linear activation function:

5 $$\begin{aligned} g(\cdot ):R\longrightarrow R, \end{aligned}$$

can be chosen from a class of functions and the weights for each layer input (depicted by the arrows to a neuron) and bias term $b_1$ parameterise the model. Both the non-linear activation functions and the weighted linear summations are chosen in order to optimise the model performance. Here, the weight $w_{1,2}$, shows the represents the respective significance of input feature 2 to the output of the neuron in the first hidden layer and $b_1$ the bias added to the weighted inputs. In many applications, most MLPs are used as variations of models with either one or two hidden layers with different number of neurons per hidden layer. Lastly, the output layer contains a logistic activation function of the form:

6 $$\begin{aligned} g(\tilde{y})= \frac{1}{\left( 1+e^{-\tilde{y}}\right) } \approx p^*, \end{aligned}$$

where $\tilde{y}=w_{L,1}y_1^{(L)}+w_{L,2}y_2^{(L)}+\ldots +w_{L,m^{(L)}}y_{m^{(L)}}^{(L)}+b_L$ and the output giving the relative probability estimate of presence^31,33^ used in the logloss function (Eq. 7). Because of the use of presence-only data, the output from Eq. (6) is relative to $p(y=1)$, see Eqs. (1) and (2) and accompanying explanation.

The MLP model is trained by minimising the log loss function using the stochastic gradient-based optimizer as described in the work by Adam et al.^30^. The most common classifier loss functions are the Area Under the Curve (AUC) function and the log loss function. The latter is chosen here because AUC is a relative measure of internal ordering, rather than an absolute measure of the quality of a set of predictions (as explained in Section 4.5 and https://www.datamachines.io/blog/auc-vs-log-loss). The model training optimises the objective function:

7 $$\begin{aligned} \min _{\textbf{W},\textbf{b}, \text {Model parameters}} \quad \frac{1}{N} \sum _{i=1}^{N} \left[ y_{i} \ln p_{i}+\left( 1-y_{i}\right) \ln \left( 1-p_{i}\right) \right] , \end{aligned}$$

where **W**,**b** are the set of weights and bias terms to be chosen, *y* is the binary true value (1 for presence, 0 for background), *p* the prediction probability (between 0 and 1, as in Eq. 2) and N is the total number of samples used for training. This logloss is minimum when low probabilities (*p*) are assigned to cells containing no presence (y=0) and high probabilities to cells containing one or more presence points (y=1). The model parameters include a number choices like activation functions, layer sizes, and optimisation hyperparameters as discussed below.

The models were implemented through the *Scikit Learn* package in Python^34^. The importance and predictive power of the several feature layers is analysed using Sklearn’s per_mutation_importance function. This shows per feature layer, its contribution to the score of the loss function (Eq. 7). More technically, the permutation feature importance is the decrease in a model score when a single feature value is randomly shuffled. To come to the relative contribution (as displayed in Fig. 4), these scores are divided by the total model score.

#### Hyperparameter optimisation

Sklearns MLPclassifier has multiple hyperparamaters including the number of hidden layers, an overfitting penalty and a number of options regarding activation functions, see Table 8. These hyperparameters are optimised using the Hyperopt package^35^. In total, 200 parameter combinations are explored with 5-fold cross-validation. For this, the model training set is split up in five equal sized parts (hence 5-fold), trained on four of these and evaluated on the remaining one. This is repeated five times such that each of the five data partitions is used once as the evaluation set. The average performance of the model in these five experiments gives an idea of how well the model will perform on data not seen during the training. These five separate models are then tested on the 30% evaluation data set, that was kept apart during training and unseen by all, and the best performing model out of the five is used to assess results, including a comparison with Max-Ent.

**Table 8:** Hyperparameters of Sklearn’s MLPClassifier and MLPRegression models. During training, 200 combinations of above parameters are tried and evaluated.

Parameter	Options
$\texttt {hidden\_layer\_sizes}$	(5,), (10,), (20,), (30,), (40,), (50,), (60,), (70,), (80,), (90,), (100,), (5,5), (10,10), (20,20), (30,30), (40,40), (50,50), (60,60), (70,70), (80,80), (90,90), (100,100)
$\texttt {activation}$	‘identity’, ‘logistic’, ‘tanh’,‘relu’
$\texttt {solver}$	’adam’
$\texttt {learning\_rate}$	‘constant’, ‘invscaling’, ‘adaptive’
$\texttt {l1\_ratio}$	0.0, 1.0
$\texttt {max\_iter}$	500, 1000, 2500

The best performing combination was used to evaluate the model. Hidden layer sizes of (50, 50) should be read as two layers with fifty nodes each, i.e. $m^{(L)}=50,$ and $L=2$ in Fig. 8.

### Estimating total number of water access points per grid cell

The most recent standard Ugandan Demographic Health Survey (DHS) is from 2016 and was conducted for the entire nation^5^, it includes information on the primary water access type of each surveyed household. From the survey data, Fig. 1 presents the distribution of the access types in the urban and rural settings in Uganda. Generally, as expected, boreholes are used both in rural and urban settings while piped access is a lot more prevalent in urban settings. However, it would be too simplistic to conclude from Fig. 1 that 50% of the rural Ugandan access point types are boreholes. The number of persons served by a water point can differ by access type^36^; for example, an on premises piped connection usually serves the household while a publicly accessible borehole is expected to serve a lot more people. In order to estimate the average number of persons that each access type serves, WPDx data can be used together with the DHS data. To show how, an example is presented in the paragraphs below.

With no prior assumptions on how many people each different access type serves in a given grid cell, and assuming that for each cell with WPDx data we have all access points, we can characterise the number of people *served by* a (WPDx) access point $s_i$ as:

8 $$\begin{aligned} s_i = \frac{Pop_i}{n_{i}}, \end{aligned}$$

where the population ($Pop_i$) and the total number of all WPDx points ($n_{i}$) (i.e. number of all access types summed) defined for each cell *i* give us the average number of users per water access point per cell. Next we can consider, per access type (boreholes in our example), the average of all the cells where presence of the target type is recorded. With no priors, the average number of people served by boreholes can then be approximated by

9 $$\begin{aligned} s_{bh} = \frac{1}{n_{bh}}\sum _{i\in X_{bh}}{} s_i, \end{aligned}$$

where $X_{bh}$ is the set of $n_{bh}$ cells where boreholes were recorded.

This gives us an estimate of the average number of people a borehole serves ($s_{bh}$). This is done separately for the urban and rural cells such that we can estimate the average number of serviced persons in urban and rural settings, $s_{bh,urban}$ and $s_{bh, rural}$, respectively. Next, by using the DHS information shown in Fig. 1 on the number of people using each access type in urban and rural settings, we can estimate the total number of persons using a borehole in the urban ($P_{bh,urban}$) and rural ($P_{bh,rural}$) settings. This is divided by $S_{bh}$ to get to the total expected number of presences of each access type in the study area:

10 $$\begin{aligned} y_{bh,j} = \frac{Pop_{bh,j}}{s_{bh,j}} \text { with }j \text { in } \{urban,\ rural\}, \end{aligned}$$

where $y_{bh,j}$ is the expected total number of borehole presences in the study area. The results for each access type are reported in Table 9 and are (well) below the maximum number of users per source reported before^36^. Potential limitations in these estimations lie in incomplete water point coverage of populated cells and the potential the non-domestic usage of some water points^36^. The fact that similar numbers of persons sharing the same access type in both urban and rural settings are found, is encouraging. This shows that there is, as one would expect, a limit on the number of persons that can access one source at the same time.

**Table 9:** Estimated average number of persons sharing one access point $s_{AT,\cdot }$ and total number of expected presences per access type $y_{AT,\cdot }$ in urban (U) and rural (R) settings, computed over the whole of Uganda.

Access type (AT)	$s_{AT,urban}$	$y_{AT, U}$	$s_{AT,rural}$	$y_{AT, R}$
Borehole	74	28857	73	303898
Packaged water	NaN	NaN	27	0
Piped water	38	64296	46	31014
Prot. shall. well	76	8446	80	40523
Prot. spring	167	5360	132	35101
Rainwater harv.	67	1848	58	11068
Surface water	39	5364	37	141120
Unp. shall. well	112	3974	159	31681
Unp. spring	107	1451	134	15448

#### Scaling the relative probability to predict number of presences

To predict the total number of expected presences, the relative probability is scaled using the total number of expected presences (described and presented in Section "Estimating total number of water access points per grid cell"). For example, for an urban cell *i*, the model output is scaled using:

11 $$\begin{aligned} y_{i}= \frac{y_{ U}}{\sum _{j=1}^{N} p^*_{j}} p^*_{i}, \end{aligned}$$

where the denominator sums over the whole *N* such cells with urban classification. For instance, to scale the relative probability output of the borehole model, then $y_i$ is the number of boreholes predicted in cell *i*, $y_{U}$ the estimated total number of boreholes in the study area cells classified as urban (Section 4.3) and $p^*_i$ the relative probability of a borehole presence per cell estimated by the models (Eq. 2). By using the information that some water access types are more present than others, it is possible to describe the variety of water access types in cells and their expected presences across the study area in approximate but absolute terms (not relative). This is an improvement compared to^11^, who use Max-Ent to predict relative probabilities presence between cells. At the same time, this improvement suggests a method to deal with one of the biggest limitations of presence-only species modeling^12^. It would unfortunately be difficult to apply this in biological species settings as estimation of the total presences might not be possible and species occurrence is (at least for animals) a lot more dynamic than static infrastructure like water access points.

### Background selection

During training, the model takes 70 % of the WPDx data as presence data, the remaining 30% is eventually used for evaluation. As the WPDx data is presence only data (see Section 4.1.2), these presence locations are compared to a randomly selected background sample that is four times the size of the WPDx data of that access type; i.e. if there are 100 presence locations, 400 background points are selected from all the cells where no presence (of all access types) is recorded. It is acknowledged that this ratio of background and presence locations is somewhat arbitrary which is why a small sensitivity analysis is presented in the Result Chapter. The used ratio was found to perform satisfactory after a quick optimisation, during which it was found that too many background points would result in overfitted data as very few locations get assigned high scores by the model, too little background points would overestimate the number of presences and allows for little distinguishing power of the model. The ideal number of background points is a subject of debate in the species modeling field and really depends of the rareness and circumstances of the species^37,38^. For now, this is considered out of scope and we will continue with the ratio of 1:4, however, this could be optimised in later work.

#### Background selection using weighted backgrounds

For a second configuration of the MLP classifier model, the presence locations are compared to background locations that are selected to be less likely to contain the specific water access type considered by the model. In Table 1 this configuration is named *NN Weighted Backg*. This way, the background locations do resemble true absence locations more than if they were selected randomly. This is done by distributing the total number of expected sources (see Section 4.3) across the study area based on the population density and the share of the population using the different sources (for urban and rural settings separately) as seen in Fig. 1. Mathematically this means:

12 $$\begin{aligned} NP_{i,pr} = P_{total} \cdot \frac{Pop_i}{\sum _{j=1}^{N}Pop_j} \end{aligned}$$

**Figure 9:**
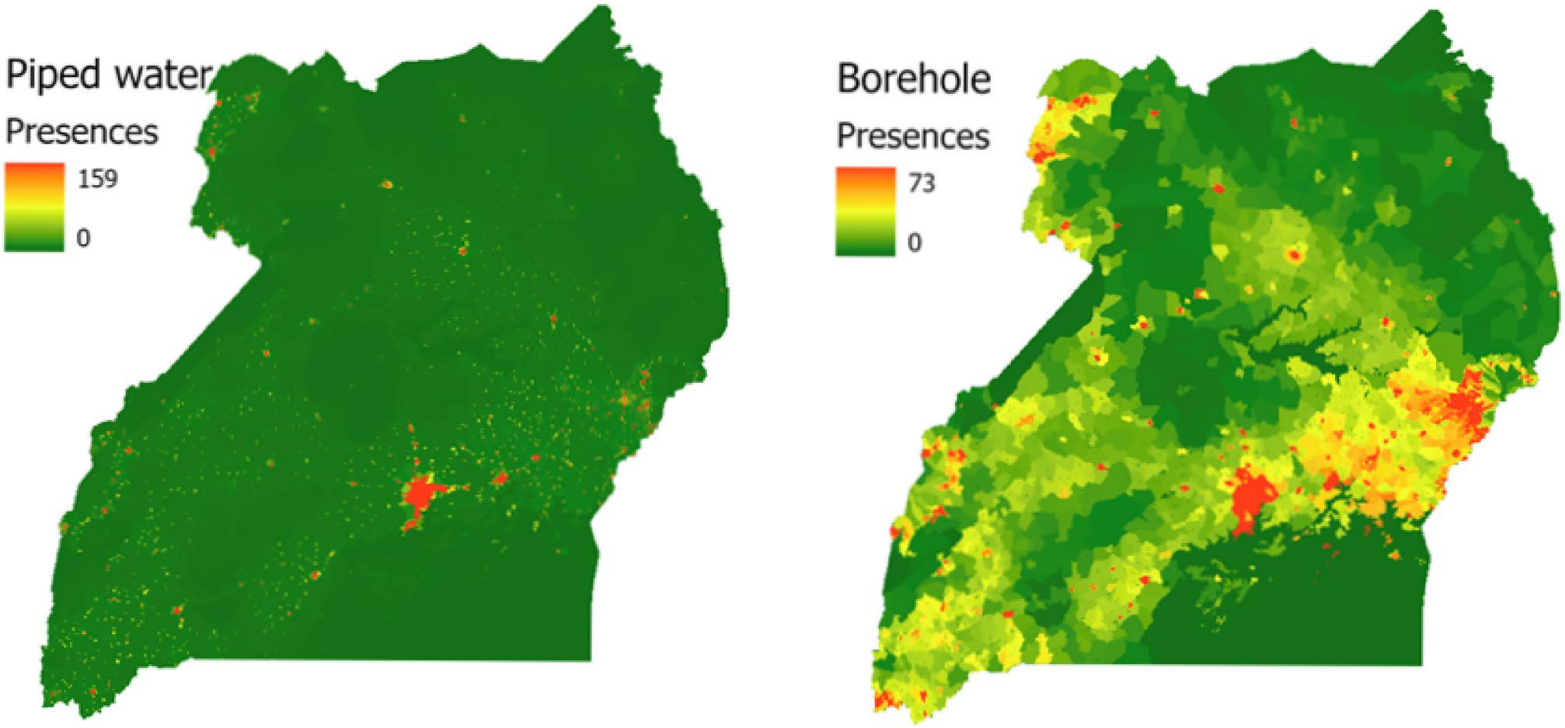
Piped water background file (left): the prior expectation of the distribution of piped water access in Uganda. Right: Borehole background file: the prior expectation of the distribution of boreholes in Uganda. Conveying with Fig. 1, Boreholes are expected to be present in both rural and urban settings, where piped water is mainly present in urban (and peri-urban) areas.

If again the example of boreholes is used, $P_{total}$ is the total number of expected borehole presences (Table 9), $Pop_i$ is the population size of cell *i*, the denominator represents the total population of all cells (= the population of Uganda) and finally, $NP_{i,pr}$ is the number of boreholes expected in cell *i* based on the population density and the share of the population using these sources in urban and rural settings (Fig. 1). By applying this to all cells and all access types, we derive a prior expectation of the distribution of the different sources, in which areas with less people are expected to have fewer water access points. This spatial distribution of expectations over grid cells will be referred to as the background files, with a different background file for each access type. Two examples can be seen in Fig. 9.

For the second model configuration (*NN Weight. Background* in Table 1), the background points are sampled randomly but with the cells selection weighted by the inverse of expected number of access points for each cell *i*:

13 $$\begin{aligned} \Omega _i = \frac{1}{NP_{i,pr}}, \end{aligned}$$

such that cells with a higher weight have a proportionally higher probability of being selected.

Cells that are expected to have zero access points (eg. for unpopulated areas) are given the highest weight of the other present cells (instead of $\infty$). Also this time, the weighted selection happens from the cells for which there is no recorded presence.

A risk with this method is that the model becomes too dependant or correlated with the population density. If only populated cells are compared to non-populated cells, the model might turn out to be only a predictor of the presence/absence of people instead of distinguishing cells with or without water access. Too dampen this effect, the population density feature layer is not included in this second model (i.e. NN Weight Backg. in Table 1). However, it is important to monitor the created bias when assessing the results.

### Validating model performance

For classification models, and especially in species modeling, the most common evaluative parameter is the Area Under the ROC Curve (AUC) operator. In short, the AUC can be seen as a comparison between the true positive rate (share of predictions exceeding threshold $\theta$ correctly) and false positive rate (a presence prediction on an absence location). It measures the ability of the model to distinguish between presence and background locations, but note that this is different from presence absence locations and therefore has different implications with respect to the traditional interpretation of the results^19,28^. In Ref.^39^, it is argued that if the objective is to predict and evaluate estimations of potential distributions, the AUC is not useful. The main reason that it is not used in this research is because the AUC places an equal weight on the true positive and false positive rate and therefore different number of background locations can cause similar AUCs but different probability distribution estimates. For example, when a high number of background points is created by the user (with binary value 0), and it is compared to a low number of presence points, the model will automatically assign a low value to most cells. This will result in low true positive rates as for most presence cells the prediction score will not exceed the threshold. For the same reason, this results in a low False Positive Rate. This is vice versa for the case where a smaller number of background locations are used compared to presence locations. As for the AUC, both the True Positive Rate and the False Positive Rate are weighed equally and with that, the AUC will not change for the number of background locations. However, the number of background locations does influence the distribution of presences, which is shown in the sensitivity analysis in Section "Modeling WPDx data" .

Therefore, instead of using the AUC, the classifying models in this work are evaluated using a presence—and false positive rate, which are introduced here. Let $l_1$ be the number of presence cells with relative probability predictions ($p^*$, Eqs. (2) and (6)) larger than a threshold $\theta$ and *n* the number of presence locations. Then the true positive rate (TPR) is defined as

14 $$\begin{aligned} \textrm{TPR} = \frac{l_1}{n}. \end{aligned}$$

Similarly, let $l_2$ be the number of background cells (no known presence) that get assigned a value larger than the same threshold $\theta$, and *s* be the number of background cells, then we define the false positive rate (FPR) as:

15 $$\begin{aligned} FPR = \frac{l_2}{s}. \end{aligned}$$

The TPR measures the ability of the model to predict presence locations given the feature data evidence, which is desired for the model. The FPR tells us whether or not a high TPR score is only there because all cells are assigned large values; therefore, it gives additional information on the distinguishing power of the model. As there are now two separate scores, both with values between 0 and 1, this allows for a better comparison of the performance of the models where one performance indicator can not make up for the other, unlike for the AUC. For the TPR the highest score of 1 implies that all presences were correctly predicted. The best score for the FPR would be 0 in a presence absence scenario, since this would mean all absence locations received lower scores than the threshold $\theta$. However in the presence-only case, some presences are expected in the background locations and so the best possible FPR in a presence only model is possibly above zero.

It is acknowledged that the choice for the threshold $\theta$ will seem arbitrary, and that access types that we know to occur less often should perhaps receive a higher threshold. However, in the absence of justifiable heuristics for assigning such varying thresholds, and since all the models receive a similar ratio of presence and background locations, a constant threshold allows here for a fair comparison between models. Furthermore, we eventually scale the **relative** probability output by the total number of presences, and so the predicted presences are not impacted by the chosen threshold value in the end.

### Survey

This survey campaign was a result of joint work between the Department of Water Management at TU Delft (Netherlands) and the Department of Environmental Management of Makerere University (Kampala, Uganda). It has the ethics approval of both the Human Research Ethics Committee (HREC) TU Delft and the Makerere University of Social Sciences Research Ethics Committee (MAKSS REC).

#### Survey design

**Figure 10:**
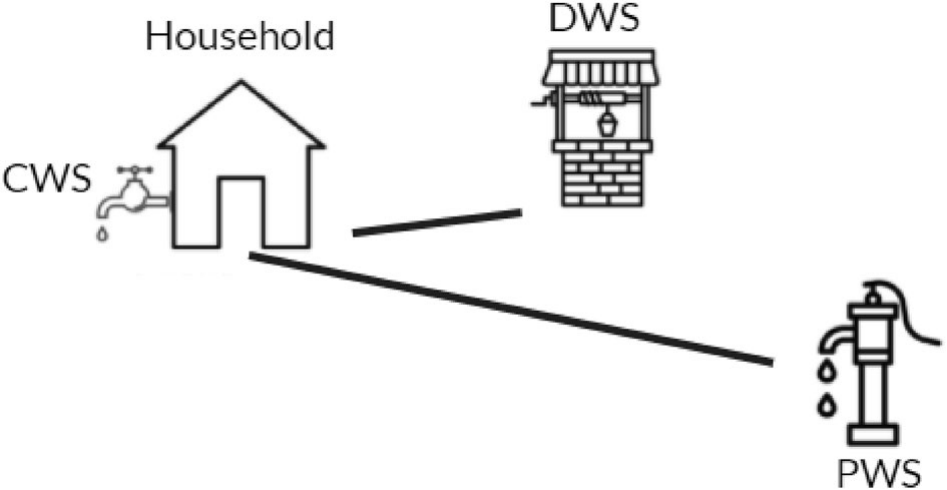
Visual of the Primary Water Source (PWS), Closest Water Source (CWS) and Drinking Water Source (DWS).

The survey consists of five sections. The first gathers information on the households size, location and economic status. The second section is on the daily total water use, the variety of used sources and the total daily travel time to and from these sources. Next, as many households in Bushenyi-Ishaka municipality make use of a variety of water sources on a daily basis^3^, a distinction is made between the Primary Water Source (PWS), the Closest Water Source (CWS) and the Drinking Water Source (DWS) (see Fig. 10). The PWS is the source the household takes the most water from for domestic use on a daily basis. On this PWS, information is gathered such as the water volume taken per trip, the travel time but also the perceived quality and purpose. Dependent on whether or not the PWS is different from the CWS and/or DWS, similar questions are asked for the CWS as well as the DWS. In that way the potentially three different sources can be compared properly. In other words: the final three sections of the survey consist of sections for the PWS and, if applicable, the CWS and DWS respectively. Perhaps superfluously, but this means that for all households information is gathered on the PWS, however, as the PWS, CWS and DWS are for many households the same, the latter two sections are often skipped. Most questions are either multiple choice, with the option to select multiple options. Sometimes, respondents are asked to fill in an integer, e.g. the daily travel time to collect water in minutes. The questions were written in English but if needed orally translated into Runyakore, which is the most widely used language in the Bushenyi district. If available, the interview was conducted with the self identified household head but if absent, another adult would suffice.

**Figure 11:**
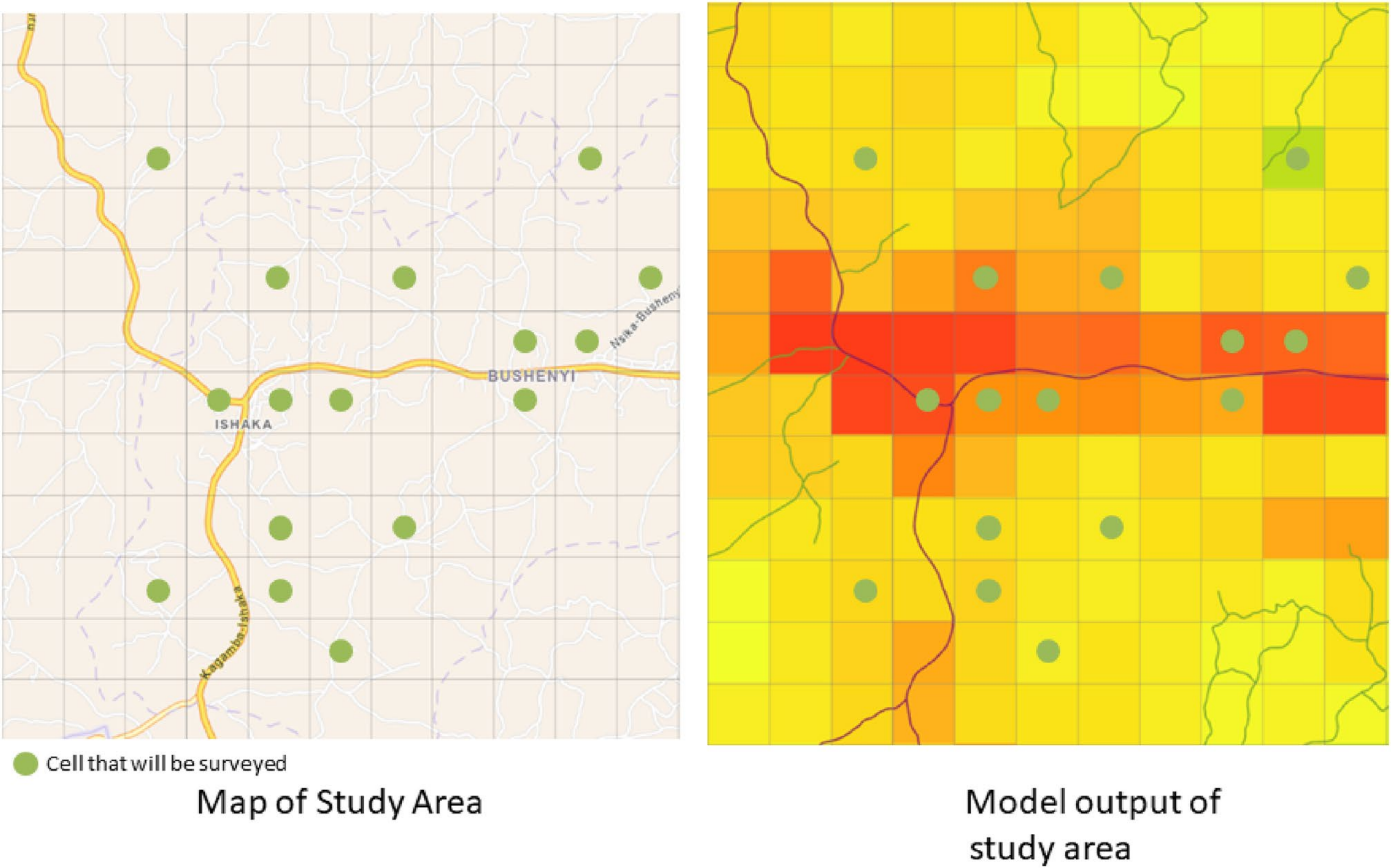
The selected cells in Bushenyi Ishaka Municipality on map (left) and summation of weighted background model outputs (right), in which red indicates many water point presences (of all types) and green little presences. Each cell is approximately 1 sq. km.

Cells were selected based on two criteria. Firstly, in discussion with local experts, cells were selected that would contain different water sources, are from both urban and rural areas and would represent households with different socio-economic status and or jobs. Secondly, the area was compared with model output such that both cells that the model would assign higher access and cells with predicted lower access, were selected. This can be seen in Fig. 11.

#### Sample size

The population size of Bushenyi district is approximately 250 thousand people (according to https://bushenyi.go.ug/district/statistics). An average household in Bushenyi consists of five persons^3^, such that the population size of interest is 50 thousand households. In order to obtain a large enough sample size *s* to be representative of the Bushenyi district, the guideline from Ref.^40^ is used:

16 $$\begin{aligned} s=\frac{\chi ^{2} N_p P_p(1-PP)}{d^{2}(N-1)+X^{2} P_p\left(1-P_p\right)} \end{aligned}$$

Where:

$\chi ^{2}=$ value of chi-square for 1 degree of freedom at the desired confidence level (= 3.841),

$N_p=$ the population size (= 50 thousand households),

$P_p=$ the population proportion (= 0.5, provides maximum sample size),

$d=$ the degree of accuracy expressed as a proportion (=.05).

The minimum representative sample size was found to be 380 households. Based on some test comparisons on mock data to find also significant results between groups and based on earlier survey campaigns in the region^3^, this was increased to a total sample size of 500 households, which is above the minimum from Ref.^40^ and therefore deemed sufficient to be a good representation.

#### Data collection

The survey was executed by a trained and experienced team of enumerators. All the enumerators also participated in the survey conducted for the study from Ref.^3^ in 2018 and thus knew the area and its dynamics well. Before interviewing the respondent, the purpose of the survey was explained and written consent was acquired. Respondents were given a unique ID that they can use to have their data removed from the database if desired. This omits the usage of names or other identifiable information. Per selected cell, approximately 30 households were surveyed, such that the total amount of interviews is around 500. Households were approached by going door to door in the selected cells. Data collection took 10 days and took place early December 2021. It is important to note that this is during one of the two Ugandan wet seasons.

The survey tool *Survey123* (https://survey123.arcgis.com/) which is a product of ArcGis, was used. The enumerators read out the questions and filled in the given answers to limit contact (Covid) and to prevent accidental misuse of the tool by respondents. The data was uploaded instantly to a secure server upon completion of the interview.

#### Survey data analysis

The survey data from Busheyni was exported to an Excel format and primarily analysed using Python. Some visualisations are made using ArcGis Pro. For comparing means and distributions of travel time and water usage datasets for different groups and access types, the following procedure is followed: First the data is tested if it represents a t-distribution using a Kolmogorov-Smirnov (KS) test. If the result indicates that one or both of the datasets (p<0.1) do not follow a t-distribution, the datasets are compared by means of a Wilcoxon rank-sum (denoted as *w*). If two datasets do follow a t-distribution, the datasets are compared by means of a student t-test. Potential correlations are tested using Pearsons rank order correlation. For comparing proportions of populations, a z-test is performed.

### Method statement

All methods were performed in accordance with the relevant guidelines and regulations. All survey respondents signed informed consent forms. No children were interviewed. The survey had the approval of both the ethics committee of TU Delft and Makerere University.

### Supplementary Information

Supplementary Information.

## Data Availability

Data will be made available upon request. Please contact Jan Geleijnse and Edo Abraham. The contact detail can be found at the beginning of this article.
