# Supplementary material for: Enhancing water access monitoring through mapping multi-source usage and disaggregated geographic inequalities with machine learning and surveys
Source: Sci Rep. 2023 Aug 18;13:13433. doi: 10.1038/s41598-023-39917-6 (PMC10439218; doi:10.1038/s41598-023-39917-6)
Supplement: Supplementary file 1 — Supplementary Information. [file 41598_2023_39917_MOESM1_ESM.pdf]

Additional information to manuscript titled:

**Enhancing Water Access Monitoring through Mapping Multi-Source Usage and Disaggregated Geographic Inequalities with Machine Learning and Surveys**


Authors:

Jan Geleijnse – Department of Water Management TU Delft

Edo Abraham – Department of Water Management TU Delft

Didier de Villiers – Department of Water Management TU Delft

James Tayebwa Bamwenda – Makerere University

# Appendix A Model output for different numbers of background points

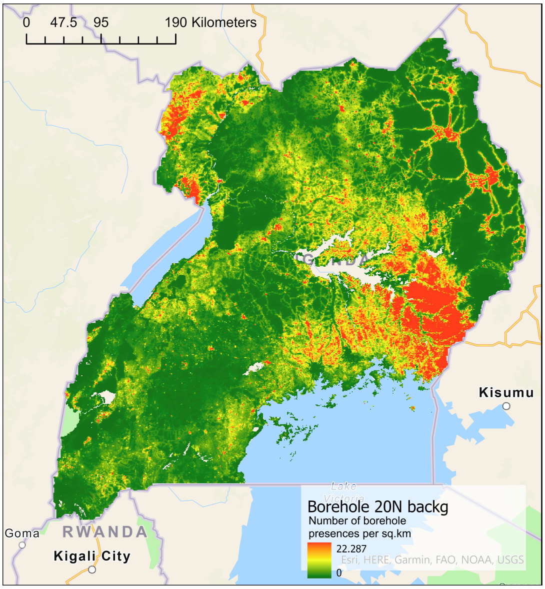


**Fig. A1**: Number of boreholes predicted by the Neural Network Standard model for 20*N number of background points in which N is the number of presences.

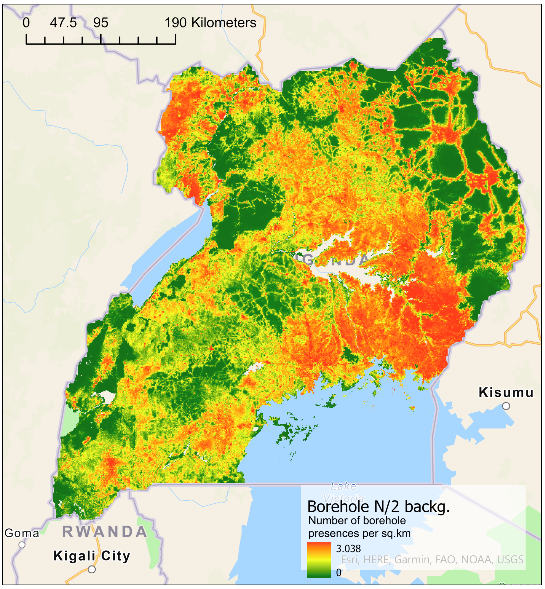


**Fig. A2**: Number of boreholes predicted by the Neural Network Standard model for N/2 number of background points in which N is the number of presences

# Appendix B Water access types used by Bushenyi households related to income

| Access Type | General access | Cat. 1 | Cat. 2 | Cat. 3 | Cat. 4 | Cat. 5 |
|---|---|---|---|---|---|---|
| Piped | 0.56 | 0.49* | 0.56 | 0.57 | 0.67* | 0.68 |
| Borehole | 0.03 | 0.01 | 0.04 | 0.02 | 0.02 | 0.09* |
| Shallow Wells | 0.28 | 0.3 | 0.23 | 0.3 | 0.28 | 0.32 |
| Springs | 0.43 | 0.38 | 0.52** | 0.44 | 0.37 | 0.27* |
| Surface Water | 0.09 | 0.05* | 0.12* | 0.1 | 0.07 | 0.05 |
| Rainwater | 0.51 | 0.43** | 0.59** | 0.52 | 0.43 | 0.59 |
| Other | 0.04 | 0.06 | 0.02* | 0.03 | 0.11** | 0.05 |

**Table B1**: Share of households that said to have access to and to regularly use the listed sources (second column). The right columns represent the same but subdivided into income categories. Significantly larger or smaller shares as compared to the total group (left column) are indicated with *p<1, **p<.05, ***p<0.01. Income categories are divided as: 0-100k SHS/month (Cat. 1), 100k-200k SHS/month (Cat. 2), 200k-500k SHS/month (Cat. 3), 500k-1M SHS/month (Cat. 4), >1M SHS/month (Cat. 5)

| Access Type | General access | No ed. | Prim. | Sec. | Tert. | Listed as PWS. |
|---|---|---|---|---|---|---|
| Piped | 0.56 | 0.45* | 0.42*** | 0.6 | 0.74*** | 0.45 |
| Borehole | 0.03 | 0.02 | 0.03 | 0.03 | 0.03 | 0.02 |
| Shallow Wells | 0.28 | 0.29 | 0.31 | 0.26 | 0.24 | 0.14 |
| Springs | 0.43 | 0.6*** | 0.44 | 0.4 | 0.4 | 0.26 |
| Surface Water | 0.09 | 0.05 | 0.1 | 0.09 | 0.06 | 0.04 |
| Rainwater | 0.51 | 0.55 | 0.44* | 0.54 | 0.53 | 0.07 |
| Other | 0.04 | 0.05 | 0.06 | 0.03 | 0.02 | 0.03 |

**Table B2**: Similar to Table B1, but this time comparing the educational level to the general access. The most right column displays the share of households that indicated the listed access type as Primary Water Source (PWS).

# Appendix C Feature layers used by model

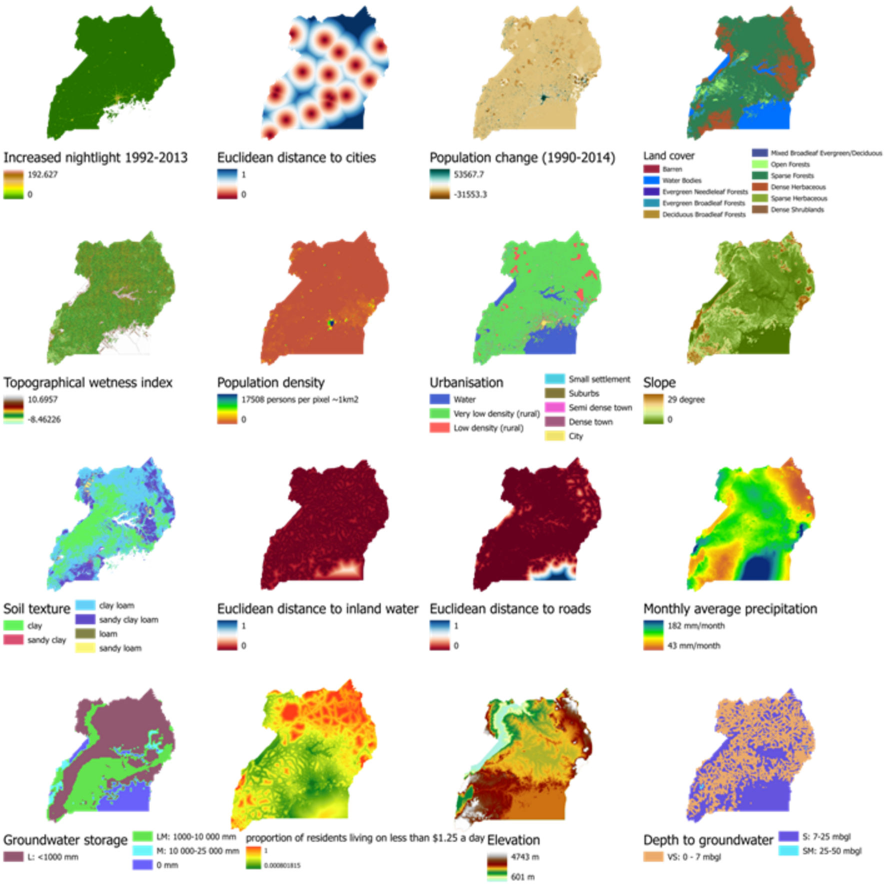


**Fig. C3**: The feature layers used by the model, also listed in Table C3

| Feature layer | Source | url |
|---|---|---|
| Increased Nightlight | SEDAC (1992-2013) | https://sedac.ciesin.columbia.edu/data/set/sdei-viirs-dmsp-dlight/docs |
| ED Cities | UNHCR(2020) | https://data2.unhcr.org/en/documents/details/85323 |
| Population Change | SEDAC (1990-2014) | https://sedac.ciesin.columbia.edu/data/set/ghsl-population-built-up-estimates-d |
| Land Cover | MODIS Land Cover Type (MCD12Ql) Version 5 (2014) | https://modis.gsfc.nasa.gov/data/dataprod/mod12.php |
| Topographical Wetness Index | ASTER GDEM Version 3 (2019) | https://earthexplorer.usgs.gov/ |
| Population Density | CEISIN (2015) | https://sedac.ciesin.columbia.edu/data/collection/gpw-v4/documentation |
| Urbanisation | Global Human Settlement Grid (2015) | https://ghsl.jrc.ec.europa.eu/ghs_smod.php |
| Slope | ASTER GDEM Version 3 (2019) | https://earthexplorer.usgs.gov/ |
| Soil Texture | ISRIC (2015) | https://data.isric.org/geonetwork/srv/eng/catalog.search#/metadata/2a7d2fb8-e0db-4a4b-9661-4809865aa |
| ED Inland Water | Digital Chart of the World (DCW) (2006) | https://www.diva-gis.org/gdata |
| ED Roads | OpenStreetMap (2021) | http://download.geofabrik.de/africa/uganda.html |
| Monthyl Average Precip. | WorldClim Global Climate Data version 1.4 (1970-2000) | https://www.worldclim.org/data/worldclim21.html |
| Groundwater Storage | Digital groundwater maps of Africa(2012) | https://www2.bgs.ac.uk/groundwater/international/africanGroundwater/mapsDownload.html |
| Poverty | Worldpop (2011) | https://www.worldpop.org/geodata/summary?id=1271 |
| Elevation | ASTER GDEM Version 3 (2019) | https://earthexplorer.usgs.gov/ |
| Depth to Groundwater | Digital groundwater maps of Africa (2012) | https://www2.bgs.ac.uk/groundwater/international/africanGroundwater/mapsDownload.html |

**Table C3**: Used feature layers and their sources.

| Water Usage In Bushenyi Survey_ Questionnaire | |
|---|---|
| **Question** | **Answer choices** |
| **Introduction** | |
| All the information you provide is confidential and your name will not be disclosed anywhere. The results will be treated anonymously. Participation in this study is voluntary. You don't have to take part if you don't want to. You don't have to answer any question you don't want to, and you can stop the interview at any time. If you decide not to participate there will not be any negative consequences.<br><br>Do you have any questions? Do you agree to participate in this study? If you have any further questions you can contact Prof. Kansiime Frank from Makerere University Department of Environment and Management at +256 772 506520 or +256 752 506520. | |
| Administer informed consent. If subject agrees to participate, proceed to questionnaire | yes, no |
| **Location of Household (GPS)** | Survey Collector can register this |
| **Personal Information** | |
| Gender of survey respondent | Female,Male |
| What is the gender of the household head? | a. Male – the respondent is head<br>b. Male – not the respondent<br>c. Female – the respondent is head<br>d. Female – not the respondent |
| How many people are in your household? | 1,2,3,4,5,More than 5 |
| What is your highest level of education completed? | • Primary education Basic level<br>• Secondary education – Ordinary level<br>• Secondary education - Advanced level<br>• Tertiary education – Vocational college<br>• Tertiary education – University degree |
| Are you able to read or write? | Yes<br>No |
| Marital status (if age is >15) | 1. Married<br>2. Living together<br>3. Unmarried/Single<br>4. Divorced<br>5. Separated<br>6. Widowed |

| | |
|---|---|
| Main occupation (if age is >15) | 1. Agriculture/Livestock/Herding<br>2. Handicrafts(Weaving)/Carpenter/Mason/Blacksmith Trader/Merchant (retail/ wholesale)/Food vendor \<br>3. Formal Employment<br>4. Casual Employment<br>5. Unemployed<br>6. Retired<br>99. Other (specify) __________ |

| | |
|---|---|
| How many people live and eat in your household on a regular basis? (Age 0 – 2, Age 3 – 5, Age 5 – 18, Age 18 or older) | Three columns: Age/Male/Female<br>Four additional rows for age breakdown: (Age 0 – 2, Age 3 – 5, Age 5 – 18, Age 18 or older) |

| **Socio-economic status** | |
|---|---|
| How much land does your family own (in Acres)? | |
| How much land do you use to cultivate vegetables or staple crops? | |
| How many of each of these animals does your household currently own? | 1. Large stock (Cattle)<br>2. Small stock (Goats/sheeps)<br>3. Pigs<br>4. Poultry |
| Does anyone in your household own the following items? (Select one or more) | 1. Radio<br>2. Mobile phone<br>3. Television<br>4. Car or truck<br>5. Hand cart<br>6. Animal<br>7. Cart<br>8. Generator<br>9. Tractor<br>10. Bicycle<br>11. Motorcycle<br>12. Solar Panel |
| Does the household have an electricity connection? | yes, no |
| How does your household afford to put food on the table? | Food/Income from Agriculture<br>Salary<br>Grants<br>Bartering<br>Allowances |
| Approximately how much income do you receive from each of these sources per month during the dry season? (In Shs) | |
| How many women in your household earn some income? | |
| About what share of household income is earned by women? | Percentage |
| Does your household receive any money from other family members living outside of this village (remittances)? | yes, no |
| About how much money do you receive from them? | |
| If you wanted to take out a loan of 10,000 Shs, from someone other than household members, could you do so? | yes, no |

| | |
|---|---|
| And in the past year, about how much did your family spend on each of the following things? | 1. Weddings<br>2. Funerals<br>3. Baptisms<br>4. Health Care (Household)<br>5. School fees (household) |

| **Waterborne disease, disability and health awareness** | |
|---|---|
| Has anyone in your household had diarrhea or respiratory illness in the last 30 days? | |
| (If yes) How many had:<br>Diarrhea (enter number): Matrix for gender / age group: 0-5; 5-18; 18-60; 60+<br>Respiratory illness (enter number): Matrix for gender / age group: 0-5; 5-18; 18-60; 60+ | |
| Which health facility is most often used by your family for health services? | Kampala International Hospital (Ishaka), Bushenyi Health Center II, Other.. |
| Have you ever heard about water borne diseases in this area? | |
| If yes in the above, which disease was it? | |
| Have you yourself had diarrhea in the past 2 weeks? | None, Once, Twice, 3 times, >3 times |
| For any of these occurrences of diarrhea, did you seek advice or treatment from any source? | Yes, hospital or health centre<br>Yes, shop of pharmacy<br>Yes, traditional healer<br>No |
| How much did you spend in total on treatment for recent diarrhea and/or respiratory illness(es) for your household, in each of these categories: Medical fees, medicines, transport to facility, Did nothing, Other | Medical fees, medicines, transport to facility, Did nothing, Other |
| Do any household members have disabilities? | Yes, no (skip) |
| How would you describe the main disability of the most disabled HH member? | 01 – Hearing impairment<br>02 – Deafness<br>03 – Visual impairment<br>04 – Blind<br>05 – Mobility impairment<br>06 – Housebound<br>07 – Upper limb impairment<br>08 – Speech impairment<br>09 – Learning difficulties<br>10 – Mental impairment |

| **Household water source/supply** | |
|---|---|
| Can you list all household water sources for both drinking and nondrinking water? | 1. NWSC/Piped<br>2. Borehole<br>3. Shallow Wells |

| | |
|---|---|
| | 4. Springs<br>5. Surface water/Rivers/stream/lake/pond/dam<br>6. Rain water<br>7. Others (Specify)------ |
| What does your household use this water for? (answer for **each** of the listed sources from previous question) Choose one or multiple | 1. Drinking<br>2. Animal use<br>3. Watering crops<br>4. Cooking<br>5. Washing<br>6. Others (Specify)……. |
| How much water does your household use on average per day throughout the year? (specify in 20 Ltr Jerry cans) | Number of 20 Ltr Jerrycans:…….. |
| How much time does your household spend on water collection per day? (**Sum** of ALL round trip(s), incl. queuing, to **ALL** sources listed) | (a) Hours ……….<br>(b) Minutes …… |
| How would you rate the cost of the water for your household? | 1. Very cheap<br>2. Inexpensive<br>3. Cost-appropriate<br>4. Expensive o Very expensive<br>5. DK/ No comment |
| Who usually fetches water in the household? | a) Man<br>b) Woman<br>c) Children<br>d) House girl/Shamba boy<br>e) Relatives<br>f) Others (Specify)….. |
| What is his/her age? | Age |
| Is that person a man or a woman | Man / Woman |
| Which of the sources listed is the source that you took the most water from during the last month? (= primary source) | 1. NWSC/Piped<br>2. Borehole<br>3. Shallow Wells<br>4. Springs<br>5. Surface water/Rivers/stream/lake/pond/dam<br>6. Rain water<br>Others (Specify)------ |
| INSERT GENERAL WATER SOURCE QUESTIONS (SEE BELOW IN YELLOW) | Questions about primary source |
| | |
| Is the primary source also the source closest to your home? | Yes / No, if **yes skip** the closest source section |
| **Closest source** | |
| What is the reason that your closest source is not your most used source? | (Allow multiple responses)<br>1=The closest water source is unsafe for drinking<br>2=The closest water source is not free or more expensive |

| | |
|---|---|
| | 3=Excluded by Water User Committee (WUC) for closest source<br>4=Excluded by user group for closest source<br>5=I don't like the taste of the closest source<br>6= Closest water source dysfunctionality<br>99= Other (specify)________ |
| Is the closest source your drinking water source? | Yes/No/Sometimes |
| INSERT GENERAL WATER SOURCE QUESTIONS (SEE BELOW IN YELLOW) | |
| Is your drinking water source the same as your primary source or closest source? | a) Same as primary<br>b) Same as closest<br>c) Same as both<br>d) No<br>If no, continue to drinking water source section. Else: skip drinking water section. |
| **Drinking water source** | |
| (If applicable) What is the reason that your drinking water source is not your closest source? | (Allow multiple responses)<br>1=The closest water source is unsafe for drinking<br>2=The closest water source is not free or more expensive<br>3=Excluded by Water User Committee (WUC) for closest source<br>4=Excluded by user group for closest source<br>5=I don't like the taste of the closest source<br>6= Closest water source dysfunctionality<br>99= Other (specify)________ |
| (if applicable) What is the reason that your drinking water source is not the same as your most used source? I.e. why do you drink from another source than the one that you use most? | (Allow multiple responses)<br>1=The most used water source is unsafe for drinking<br>2=The most used water source is not free or more expensive<br>3 = The drinking water source I use, is better tasting<br>99 = Other (specify) _____ |
| INSERT GENERAL WATER SOURCE QUESTIONS (SEE BELOW IN COLOR) | Questions about drinking water source |
| In the last four weeks, how often did it happen that you wanted to drink water, but you forgot to treat it in time? | In the last four weeks, how often did it happen that you wanted to drink water, but you forgot to treat it in time? |
| | |
| **GENERAL WATER SOURCE QUESTIONS** | This section should be inserted at all places indicated (max 3 times) |
| Select section: (for administration purposes, enumerator can fill this question in) | Primary source / closest source / drinking water source |

| | |
|---|---|
| Geo-reference: water source | Enumerator to record GIS coordinates |
| Is that source available during the entire year? | (a) No only in the wet season<br>(b) No, only in the dry season<br>(c) No, it is often broken<br>(d) Yes |
| Is water from this source safe to drink? | a) Safe to drink<br>b. Safe to drink after treatment<br>c. Unsafe to drink |
| How do treat you your water from this source before use or drinking? | a) Boiling,<br>b) Take directly from source<br>c) Using water guard,<br>d) I do not treat the water<br>e) Others (Specify)….. |
| Is this source protected from - or monitored for potential contamination? | Yes/no |
| On average, how many 20 ltr jerrycans do you take back home when using this source? | Number of 20 ltr jerrycans |

| | |
|---|---|
| On average, how long does it take to collect water from this water source? (Round trip, incl. queuing) | (a) Hours …….<br>(b) Minutes ….. |
| How do you travel to this water source? | a. By walking<br>b. By Bicycle<br>c. By Motor bike<br>d. By Car<br>e. Other: _____________ |
| Is this water source shared with other households? | a) Yes<br>b) No |
| Do you pay for water from this water source? If yes, how much do you pay ………. /20ltr jerrycan | a. Price: ____ (fill in total price)<br>b. Nothing<br>c. I don't know |
| Whom do you pay for water? | 1=Local government<br>2=Utility company Standpipe manager<br>3=Tanker truck manager<br>4=Water vendor<br>5=Neighbor<br>Others (Specify)……… |
| Who is responsible for managing and maintaining the main drinking water source? | 1=WUC<br>2=Community members<br>3=Person hired by the community<br>4=NWSC<br>77=Do not know<br>99=Other (specify)_____ |
| What restrictions are there, if any, to use this water source? | 1= YES, Resource contribution for scheme construction<br>2=YES, Resource contribution for repair and maintenance<br>3=YES, Membership in WUC<br>4= YES, Payment for water use<br>99= Other (specify)______<br>2=NONE |
| What is your perception about quality of water from this water source? | 1=Very Bad<br>2=Bad<br>3=Reasonable<br>4=Good<br>5=Very Good<br>77=Don't know |
| If very bad/bad, can you explain why you think this? | **Select all that apply:**<br>1=Water is salty<br>2=Smells bad<br>3=Tastes bad<br>4=Water is muddy |

| | |
|---|---|
| | 5=Contaminated by animals<br>6=Iron taste<br>99=Other (specify) ___________ |
| Has this water source experienced any service interruptions over the past 3 months (interruption = no water available for 12 hours or more)? | a. Yes<br>b. No |
| (If yes) How long was the service interrupted (enter hours, days or months)? | |
| (If yes) What was the main cause of the *interruption in service?* | |
| Is this water source currently functional? | Yes/No |
| If NOT, why is it not functional? | |
| Do you think this water source will be operating in 5 years? | |
| If main drinking water source needed repairs, how confident are you that the problem could be fixed within 1 week? | |
| In the last 6 months, were there any times when water from main drinking water source was not available for more than one week? | |
| On a scale of 1 to 10, how do you rate the following aspects of water services:<br>(a) Regular or continuous supply;<br>(b) Cleanliness;<br>(c) Safety (Public Health);<br>(d) Safety (Physical) | Likert scale |
